# Modeling ethanol production through gas fermentation: a biothermodynamics and mass transfer-based hybrid model for microbial growth in a large-scale bubble column bioreactor

**DOI:** 10.1186/s13068-020-01695-y

**Published:** 2020-03-27

**Authors:** Eduardo Almeida Benalcázar, Henk Noorman, Rubens Maciel Filho, John A. Posada

**Affiliations:** 1 Department of Product and Process Development, Faculty of Chemical Engineering, State University of Campinas, Av. Albert Einstein 500, Cidade Universitária, Campinas, SP 13083-852 Brazil; 2 Department of Biotechnology, Faculty of Applied Sciences, Delft University of Technology, Van der Maasweg 9, 2629 HZ Delft, The Netherlands; 3 DSM Biotechnology Center, A. Fleminglaan 1, 2613 AX Delft, The Netherlands

**Keywords:** Ethanol, Bioreactor simulation, Biothermodynamics, Syngas fermentation

## Abstract

**Background:**

Ethanol production through fermentation of gas mixtures containing CO, CO_2_ and H_2_ has just started operating at commercial scale. However, quantitative schemes for understanding and predicting productivities, yields, mass transfer rates, gas flow profiles and detailed energy requirements have been lacking in literature; such are invaluable tools for process improvements and better systems design. The present study describes the construction of a hybrid model for simulating ethanol production inside a 700 m^3^ bubble column bioreactor fed with gas of two possible compositions, i.e., pure CO and a 3:1 mixture of H_2_ and CO_2_.

**Results:**

Estimations made using the thermodynamics-based black-box model of microbial reactions on substrate threshold concentrations, biomass yields, as well as CO and H_2_ maximum specific uptake rates agreed reasonably well with data and observations reported in literature. According to the bioreactor simulation, there is a strong dependency of process performance on mass transfer rates. When mass transfer coefficients were estimated using a model developed from oxygen transfer to water, ethanol productivity reached 5.1 g L^−1^ h^−1^; when the H_2_/CO_2_ mixture is fed to the bioreactor, productivity of CO fermentation was 19% lower. Gas utilization reached 23 and 17% for H_2_/CO_2_ and CO fermentations, respectively. If mass transfer coefficients were 100% higher than those estimated, ethanol productivity and gas utilization may reach 9.4 g L^−1^ h^−1^ and 38% when feeding the H_2_/CO_2_ mixture at the same process conditions. The largest energetic requirements for a complete manufacturing plant were identified for gas compression and ethanol distillation, being higher for CO fermentation due to the production of CO_2_.

**Conclusions:**

The thermodynamics-based black-box model of microbial reactions may be used to quantitatively assess and consolidate the diversity of reported data on CO, CO_2_ and H_2_ threshold concentrations, biomass yields, maximum substrate uptake rates, and half-saturation constants for CO and H_2_ for syngas fermentations by acetogenic bacteria. The maximization of ethanol productivity in the bioreactor may come with a cost: low gas utilization. Exploiting the model flexibility, multi-objective optimizations of bioreactor performance might reveal how process conditions and configurations could be adjusted to guide further process development.

## Background

Gas mixtures containing CO_2_, H_2_ and CO are commonly known as syngas, which is typically produced by two processes, i.e., thermochemical conversion (gasification) of carbonaceous materials like coal and oil, and reforming of natural gas [1]. Lignocellulosic biomass, food, municipal and packaging wastes are alternative raw materials that can also be used for gasification [2, 3] and could lead to production processes with improved sustainability attributes as compared to fossil-based feedstocks [4, 5]. For this reason, syngas from non-fossil sources is considered a key feedstock for the circular economy.

Driven by technical and sustainability limitations of Fischer–Tropsch synthesis [6], experiments performed since the late 1980s explored the potential of certain types of autotrophic acetogenic bacteria to catabolize the three main components of syngas into ethanol [7]. Although with generally low productivities, these microorganisms are also able to produce a variety of other substances of commercial importance, e.g, 2,3-butanediol, butanol and butyric and lactic acids [8, 9]; however ethanol is the first commercialized bioproduct [7].

Acetogens convert carbon into acetyl-CoA through the Wood–Ljungdahl metabolic pathway (WLP) [10]. In the reductive direction, the WLP is considered the most efficient non-photosynthetic and the only linear CO_2_ fixation pathway to acetyl-CoA [1, 11]. Two molecules of CO_2_ and/or CO are fixated following two separate branches, the methyl (eastern) and the carbonyl (western) branches. Thorough descriptions on the configuration of the WLP and its link with the particular energy conservation strategies of acetogens can be found elsewhere [1, 9, 12, 13]. The WLP is able to use CO as a source of energy and carbon [14–17], whereas H_2_ has to be combined with a carbon source that can be CO_2_ [9, 18]. It has been proposed that CO fermentation would yield higher amounts of Gibbs free energy and adenosine triphosphate (ATP) than H_2_ [19, 20], while H_2_ fermentation offers advantages on improved mass transfer due to higher solubilities and diffusion rates than those for CO [20]. Yet, the influence of the gas composition on the technical, economic and environmental performances of the fermentation process still remains quantitatively uncertain, basically due to the inaccuracy of currently available models of the metabolism of acetogenic bacteria.

Several types of mathematical models have been proposed for understanding and predicting the behavior of microorganisms in gas fermentations [21–27]; other simpler models have been used for estimating process performance [4, 5, 28–31]. The most popular of the modeling strategies employed recently by researchers is the genome-scale modeling (GSM), which has been used for assessing several features of the intracellular processes in *C. ljungdahlii* and *C. autoethanogenum* during syngas fermentations, e.g, the influence of the link between energy conservation and carbon metabolism on the selectivity between ethanol and acetic acid [25, 32–34], the co-factor specificity of certain enzymes linked to energy conservation [32, 33, 35], the formation of biofilms [26], the possibility of boosting ATP production by supplying arginine [36], and the feasibility of gene knock-out to reach overproduction of native and non-native products of acetogens [37]. Alternatively, with issues generally regarding on the accuracy of the quantitative predictions, GSM has also been used to assess the behavior of simulated microorganism inside large-scale bioreactors [21, 26, 37, 38]; the main cause for these latter issues may be credited to the interlinking between the intracellular processes and the environmental conditions given by the bioreactor, besides GSM’s large dependency on the objective function and the constraints applied to solve the intracellular rates of reaction [25]. The low detail of intracellular kinetics is viewed as another limitation of GSM [39] that becomes relevant given the fact that microorganisms do not reach steady-state inside large-scale bioreactors [40].

Moreover, in most publications reporting models of gas fermentations, scarce effort was invested on comparing the simulation results with experimental data reported by other research groups. Such task is challenging considering the large variety of microbial strains, gas compositions, process conditions used in the reported experiments and the high strain-specificity of more complex models of microbial metabolism. Thus, a general model that focuses on the basic thermodynamic interactions driving the catabolism of CO, CO_2_ and H_2_ by a hypothetical strain of acetogenic bacteria (such as in [28]) might be able to consolidate the diversity of reported results.

On the same line, the present study focuses on reinforcing the quantitative aspects of a previously published model [4] by validating the stoichiometric and kinetic parameters of microbial reactions with data and observations reported in scientific literature. The model is then applied to the simulation of an industrial ethanol production case and used to assess the influence of dissolved gas concentrations on bioreactor performance, gas flow profiles, supported accumulation of cells and energy requirements of the fermentation plus downstream processing of the alcohol. The model is intended to be sufficiently flexible and accurate that its results could guide further process design and optimization through model-based scaled-down experiments [41]. Finally, the model construction scheme here presented could be adapted to other process configurations, modes of fermentation and after further refining, coupled to GSM’s, intracellular kinetics and Euler–Lagrange modeling strategies [42].

## Results and discussion

This section begins with an assessment of the estimations delivered by the thermodynamics-based black-box model of microbial reactions; the analysis focuses on the predictions’ quantitative reliability by comparing the estimations with data and observations reported in literature for microorganisms that perform similar metabolic reactions. Then the analysis is extended to the characterization of two bioreactor operation regimes in terms of gas flow profiles, supported biomass accumulations, restrictions suggested by thermodynamic feasibility of catabolic reactions at different heights of the bioreactor and finally, process performance. The analysis is lastly closed with the influence of the kinetic parameters on process performance.

### Analysis of black-box model of microbial reactions

#### Gibbs free energy change of catabolic reactions

**Fig. 1:**
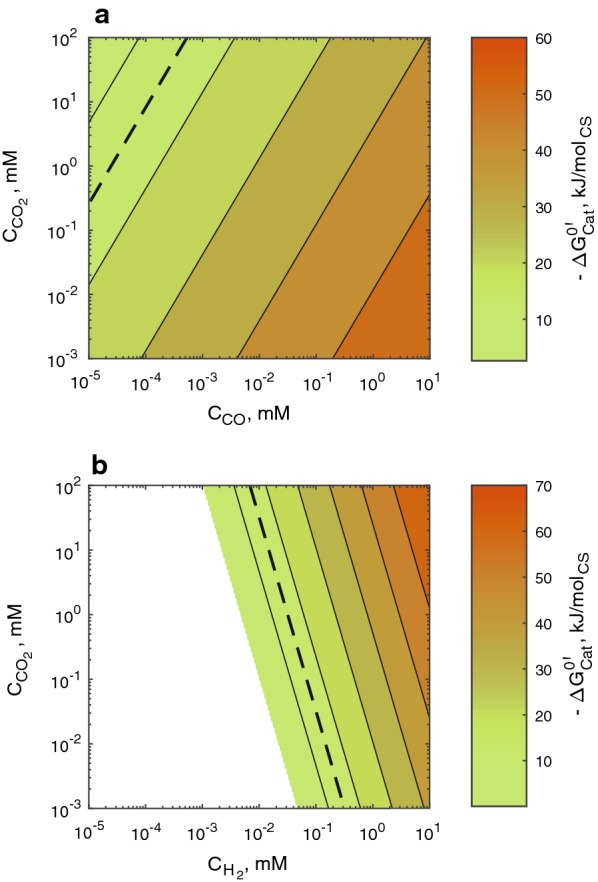
Gibbs free energy generation through independent a) CO and b) H_2_/CO_2_ catabolism for ethanol production. The dashed lines indicate where $\Delta G_{\text{cat}}^{{0^{\prime}}}$ = – 15 kJ mol_CS_^−1^. H_2_/CO_2_ catabolism is cut-off at $\Delta G_{\text{cat}}^{{0^{\prime}}}$ = 0, where the catabolic reaction would be at equilibrium and no energy could be released from it; the white region represents the gas concentrations where the inverse reaction (H_2_/CO_2_ production from ethanol) would be spontaneous

Figure 1 shows the dependence of the Gibbs free energy change in the catabolic reactions ($\Delta G_{\text{cat}}^{{0^{\prime}}}$) (see Eqs. 1 and 2 in Table 1) on dissolved gas concentrations; results are presented for independent catabolism of CO and H_2_/CO_2_ in Fig. 1a and b, respectively. They show that at dissolved concentrations of the electron donors ($C_{D}$) lower than 1 mM, the amount of energy harvested from CO catabolism is larger than that from H_2_/CO_2_ catabolism. Consequently, H_2_ threshold concentrations ($- \Delta G_{\text{cat}}^{{0^{\prime}}}$ = 9.1–15 kJ mol^−1^) for catabolic ethanol production fall between 3 × 10^−3^ and 3 × 10^−1^ mM and lower than 4 × 10^−4^ mM for CO, depending on the CO_2_ concentration ($C_{{{\text{CO}}_{ 2} }}$). Increasing ($C_{{{\text{CO}}_{ 2} }}$) diminishes the amount of energy harvested from CO catabolism where CO_2_ is a product; whereas $C_{{{\text{CO}}_{ 2} }}$ is beneficial for energy production in H_2_/CO_2_ catabolism where CO_2_ is the carbon source (see Eqs. 1 and 2).

**Table 1:** Catabolic reactions leading to the production of ethanol and acetate and related standard changes in Gibbs free energy and enthalpy

Reaction^a^	$\Delta G_{\varvec{r}}^{0}$		$\Delta H_{\varvec{r}}^{0}$		Eq nr.
	kJ mol_CS_^−1^	kJ mol_P_^−1b^	kJ mol_CS_^−1^	kJ mol_P_^−1b^	
$- 6{\text{CO}} - 3{\text{H}}_{2} {\text{O}} + {\text{C}}_{2} {\text{H}}_{5} {\text{OH}} + 4{\text{CO}}_{2}$	− 37.4	− 224.4	− 57.4	− 344.0	(1)
$- 6{\text{H}}_{2} - 2{\text{CO}}_{2} + {\text{C}}_{2} {\text{H}}_{5} {\text{OH}} + 3{\text{H}}_{2} {\text{O}}$	− 52.3	− 104.6	− 178.8	− 357.6	(2)
$- 4{\text{CO}} - 2{\text{H}}_{2} {\text{O}} + {\text{C}}_{2} {\text{H}}_{3} {\text{O}}_{2}^{ - } + {\text{H}}^{ + } + 2{\text{CO}}_{2}$	− 33.6	− 134.3	− 65.0	− 260.0	(3)
$- 4{\text{H}}_{2} - 2{\text{CO}}_{2} + {\text{C}}_{2} {\text{H}}_{3} {\text{O}}_{2}^{ - } + {\text{H}}^{ + } + 2{\text{H}}_{2} {\text{O}}$	− 28.1	− 56.2	− 134.7	− 269.5	(4)

^a^The stoichiometry of catabolic reactions and the energy changes are defined to satisfy balances on all elements involved, charge and degree of reduction. Standard Gibbs free energy and enthalpy of formation of the compounds involved in Eqs. (1–4) were retrieved from the supplementary material in [43]

^b^Results are expressed per mole of product, i.e., the product in Eqs. 1 and 2 is ethanol while acetate is the product in Eqs. 3 and 4

Neither CO nor H_2_ threshold concentrations have been reported for acetogens during solventogenesis; however, there are reports for acetogens during acetogenesis. In one of these reports, CO uptake (see Eq. 3) by *Carboxydothermus hydrogenoformans* at 65 °C stopped at a CO partial pressure ($p_{\text{CO}}$) of 3.9 × 10^1^ Pa when CO_2_ was allowed to accumulate in the overhead (reaching 1.3 × 10^5^ Pa); when CO_2_ was instead withdrawn from the overhead ($p_{{{\text{CO}}_{2} }}$ of 3.5 × 10^2^ Pa), CO uptake stopped at $p_{\text{CO}}$ of 2.0 × 10^−1^ Pa [44]. At these two points, $\Delta G_{\text{cat}}^{{0^{\prime}}}$ estimated with Eq. (5) is − 21 and − 15 kJ mol_CS_^−1^, respectively (see Additional file 1: Figure S1); intracellular acetate concentration is assumed at 10 mM and the total pressure is 2 × 10^5^ Pa. Similarly, another report mentions that *Acetobacterium woodii* started growing on H_2_ and CO_2_ at 30 °C only after $p_{{{\text{H}}_{ 2} }}$ was higher than 2.5 × 10^2^ Pa while $p_{{{\text{CO}}_{2} }}$ was 2.0 × 10^4^ Pa [45]. Assuming *A. woodii* H_2_/CO_2_ catabolism followed Eq. (4), $\Delta G_{\text{cat}}^{{0^{\prime}}}$ is estimated by Eq. (5) at − 17.9 kJ mol_CS_^−1^ (see Additional file 1: Figure S2); the intracellular acetate concentration is assumed at 10 mM and the reported total pressure is 1 × 10^5^ Pa. This brief analysis shows that Eq. (5) may be used to predict threshold concentrations for acetate production from gas fermentations with an acceptable level of approximation. Therefore, since energy conservation in acetogenic bacteria is possible during solventogenesis [19], then threshold concentrations might as well be predicted by Eq. (5) for catabolic ethanol production from CO and H_2_:

5 $$\Delta G_{r}^{{0^{\prime}}} = \left[ {\frac{{\Delta G_{r}^{0} }}{298.15} + \Delta H_{r}^{0} \cdot \left( {\frac{1}{T} + \frac{1}{298.15}} \right)} \right] \cdot T + {\mathcal{R}} \cdot T \cdot \mathop \sum \limits_{j = 1}^{m} \nu_{j}^{r} \cdot \ln C_{j} .$$

The importance of predicting threshold concentrations relies on the fact that the large-scale bioreactor should be designed to avoid reaching such concentrations.

#### Biomass yields

Since biomass yields ($Y_{{x/{\text{CS}}}}$) depend on $\Delta G_{\text{cat}}^{{0^{\prime}}}$ (see Eq. 6) they are a direct function of $C_{D}$ and follow a similar trend as $\Delta G_{\text{cat}}^{{0^{\prime}}}$ when plotted against $C_{{{\text{CO}}_{ 2} }}$ and $C_{D}$ (see Additional file 1: Figure S3). Biomass yields for CO catabolism are estimated between 0.022 and 0.080 Cmol_*x*_ mol_CS_^−1^ which are slightly higher than those estimated for H_2_/CO_2_ (0.015–0.067 Cmol_*x*_ mol_CS_^−1^) mainly due to the larger amounts of Gibbs free energy dissipated by cells growth using CO_2_ as CS (see Eq. 2).

6 $$\frac{1}{{Y_{{x/{\text{CS}}}} }} = \frac{{\Delta G_{\text{dis}} }}{{\Delta G_{\text{cat}}^{0'} }} + \frac{{\gamma_{x} }}{{\gamma_{D} \cdot \nu_{D}^{\text{an}} }}$$

Similar to threshold concentrations, biomass yields have not been reported for gas fermentations during solventogenesis. However, there are specific rates of CO and H_2_ consumption reported for continuous fermentations at steady-state [32, 33, 46] that can be used to estimate biomass yields. Since in those reports, CO and H_2_ were simultaneously consumed whereas CO_2_, acetic acid and ethanol were produced, the biomass yield is estimated by dividing the dilution rate (assuming the rate of cell lysis is negligible) by the reported specific CO uptake rate ($q_{\text{CO}}$). The estimated biomass yields range between 0.044 and 0.090 Cmol_*x*_ mol_CS_^−1^ (the specific data used for his calculation is shown in Additional file 1: Table S1) which are slightly higher (yet, within the same order of magnitude) than the estimations given by Eq. (6) for ethanol catabolic production.

#### Assessment of kinetic parameters

##### Maximum specific substrate uptake and growth rates

Regarding the predicted kinetic parameters, the thermodynamics-based black-box model returns a maximum substrate uptake rate ($q_{D}^{ \text{max} }$) of −  4.4 mol_D_ Cmol_*x*_^−1^ h^−1^ (see Eq. 7) for both catabolic energy sources, CO and H_2_. The result is the same for both electron donors since they have the same degree of reduction (2 mol_*e*_− mol_D_^−1^). In addition, as explained in section “Methods”, the maximum consumption and production rates of all compounds involved in the microbial reactions are estimated by linearly relating the predicted stoichiometry with the maximum substrate uptake rates. As consequence, the maximum growth rate ($\mu^{ \text{max} }$) was estimated at 0.29 and 0.19 h^−1^ for CO and for H_2_/CO_2_ fermentations, respectively. $\mu^{ \text{max} }$ for H_2_/CO_2_ fermentation is two times lower than CO fermentation because although $q_{D}^{ \text{max} }$ is the same on both cases, $y_{{x/H_{2} }}$ (per mole of electron donor) for H_2_/CO_2_ fermentation is also two times lower than the $y_{x/CO}$ (see Eqs. 1–4 and Additional file 1: Figure S3).

7 $$q_{D}^{ \text{max} } = 3 \cdot { \exp }\left[ {\frac{ - 69000}{{\mathcal{R}}} \cdot \left( {\frac{1}{T} + \frac{1}{298.15}} \right)} \right] \cdot \left( {\frac{1}{{\gamma_{D} }}} \right)$$

Mohammadi et al. [47] calculated a $q_{\text{CO}}^{ \text{max} }$ and a $\mu^{{{ \text{max} } }}$ of − 0.87 mol_D_ Cmol_*x*_^−1^ h^−1^ (assuming the same molar mass for cell material as here) and 0.195 h^−1^, respectively. $q_{\text{CO}}^{ \text{max} }$ was estimated by fitting batch dissolved CO concentrations (calculated using a method similar to [48]) into the kinetic equation for CO uptake (see section “Thermodynamics-based black-box model of microbial reactions”), while $\mu^{{{ \text{max} } }}$ was found by fitting batch growth data from the same experiment. In that study, syngas with a H_2_/CO ratio of 1 was used and H_2_ consumption was acknowledged; however $q_{\text{CO}}^{ \text{max} }$ was calculated without accounting for the electrons taken up from H_2_ and the carbon from CO_2_. If H_2_ and CO_2_ uptake would have been considered, the maximum electron uptake rate would be twice as large as reported, i.e., − 1.74 mol_D_ Cmol_*x*_^−1^ h^−1^. That value is 2.5 times lower than that predicted by Eq. (7). In addition, their reported $\mu^{{{ \text{max} } }}$ is close to the value estimated with the black-box model. Thus, it could be argued that *C. ljungdahlii* has a 60% reduced electron uptake capacity compared to the maximum estimated for *E. coli* [49]; yet, more steady-state data with different carbon sources is needed to confirm such conclusion. Accounting for this uncertainty, the bioreactor simulation is performed using the value estimated with Eq. (7) and the influence of a reduced maximum uptake rate on the operation of the gas fermentor is explored as part of the sensitivity analysis (see section “Sensitivity analysis”).

In addition, since growth rates estimated by genome-scale reconstructions of *C. ljungdahlii* and *C. autoethanogenum* [25, 37] used the same $\mu^{\text{max }}$ value reported by [47], their estimations are also comparable with those made by the biothermodynamics-based black-box model (see Additional file 1: Figure S4).

##### CO and H_2_ half-saturation constants

A value of 1.7 × 10^−2^ mM has been calculated for the CO half-saturation constant in ($K_{\text{CO}}$) *C. ljungdahlii* from CO consumption curve fitting [47], whereas $K_{{{\text{H}}_{ 2} }}$ has been estimated to range between 4 × 10^−2^ and 3 × 10^−1^ mM from assays using enzymatic extracts from acetogens [50–53]. In nature, wetland peats and marine waters oxidize CO with $K_{\text{CO}}$ values ranging from 1 × 10^−6^ to 4 × 10^−5^ mM [54, 55], while the averaged CO concentration in Earth’s troposphere is equivalent to a concentration of 5 × 10^−7^ mM in pure water [56]. Similarly, $K_{{{\text{H}}_{ 2} }}$ for H_2_ consumption by soils and methanogenic sludge has been estimated between 7 × 10^−8^ and 1 × 10^−6^ mM [57, 58], while the equivalent saturation from H_2_ concentration in the troposphere is 4 × 10^−7^ mM [59].

As consumption of 1 mol of CO results in higher Gibbs free energy gains than 1 mol of H_2_, it could be postulated that cells in nature control metabolic activity at low dissolved gas concentrations by stimulating H_2_ uptake (with higher affinity, $K_{{{\text{H}}_{ 2} }}$ < $K_{\text{CO}}$). This argument is in accordance with the affinities by which microbes consume H_2_ and CO in nature. However, most H_2_ consumption studies have focused on methanogens. H_2_ threshold concentrations for catabolic methane production (see Eq. 8) can be as low as 5 × 10^−6^ mM, assuming pre-industrial atmospheric concentrations for CO_2_ and CH_4_ (230 ppm and 540 ppb, respectively [60]) and using Eq. (5). Moreover, evidence suggests that acetogens may promote H_2_ production (from $H^{ + }$ ions) to avoid harmful concentrations of reduced energy carriers when feeding on CO [33, 61]. Therefore, $K_{{{\text{H}}_{ 2} }}$ might not necessarily be lower than $K_{\text{CO}}$, in agreement with the ranges of H_2_ and CO threshold concentrations estimated for ethanol production (see section “Gibbs free energy change of catabolic reactions”) and reported data for acetogens.

8 $$- 4{\text{H}}_{2} - {\text{CO}}_{2} + {\text{CH}}_{4} + 2{\text{H}}_{2} {\text{O}} .$$

It has been argued that half-saturation constants of poorly soluble substances can be overestimated by two orders of magnitude if they are derived from the fitting of consumption curves obtained under mass transfer or other rate limitations [58]. Therefore, the $K_{{{\text{H}}_{2} }}$ and $K_{\text{CO}}$ values of 4 × 10^−2^ and 5 × 10^−3^ mM, respectively, were randomly picked aiming to reconcile the information reported in literature (which is prone to overestimation) with the threshold concentrations criteria. Thus, the value for $K_{\text{CO}}$ falls midway between the estimated threshold range (see Fig. 1) and the reported value for *C. ljungdahlii*, while $K_{{{\text{H}}_{2} }}$ is located in the middle of the threshold range while simultaneously agreeing with the values determined from enzymatic extracts. Nevertheless, the effect of the value of substrate half-saturation constants on the operation of the gas fermentor is discussed in detail as part of the sensitivity analysis (see section “Sensitivity analysis”).

#### Main limitation of the black-box model of microbial reactions

Since the black-box model of microbial reactions is based on the electron transfer from one electron donor to one electron acceptor, it is not compatible with the simultaneous uptake of more than one electron donor or the generation of more than one product. Thus, the documented influence of process conditions such as pH [46, 62], acetic acid concentration [63], gas compositions [33] and gas dissolved concentrations [32] on the selectivity for either electron donor or for the production of ethanol and acetic acid, could not be reproduced. To perform such analysis, the black-box may be opened and include the mechanisms by which cells adjust the amounts of Gibbs free energy used for ATP production, depending on the specific requirements for growth, maintenance, transport of metabolites across the membrane and motile functions [49, 64, 65].

### Analysis of mass transfer-based model of the large-scale bioreactor

#### Basis of analysis

Although the algorithm that links the black-box model of the microbial reactions with the mass transfer-based model of the large-scale bioreactor uses $C_{\text{CO}}$ and $C_{{{\text{H}}_{2} }}$ as independent variables, dissolved gas concentrations are not independent during bioreactor operation. Moreover, within this section bioreactor performance is discussed from the perspective of a non-dimensional specific uptake rate of the electron donors ($q^{\prime}_{D}$, see Eq. 9) in order to partly bypass the uncertainties related to the value of $q_{D}^{ \text{max} }$:

9 $$q^{\prime}_{D} = \frac{{q_{D} }}{{q_{D}^{ \text{max} } }}.$$

The dependence of ethanol productivity and gas utilization on $q^{\prime}_{D}$ reveals the existence of two operational regimes of the bioreactor at steady-state: (i) one where mass transfer is suboptimal and (ii) one where mass transfer is sufficient. Sections “The ‘suboptimal’ operation regime” and “The ‘optimal’ operation regime” describe the features of each regime.

#### The ‘suboptimal’ operation regime

**Fig. 2:**
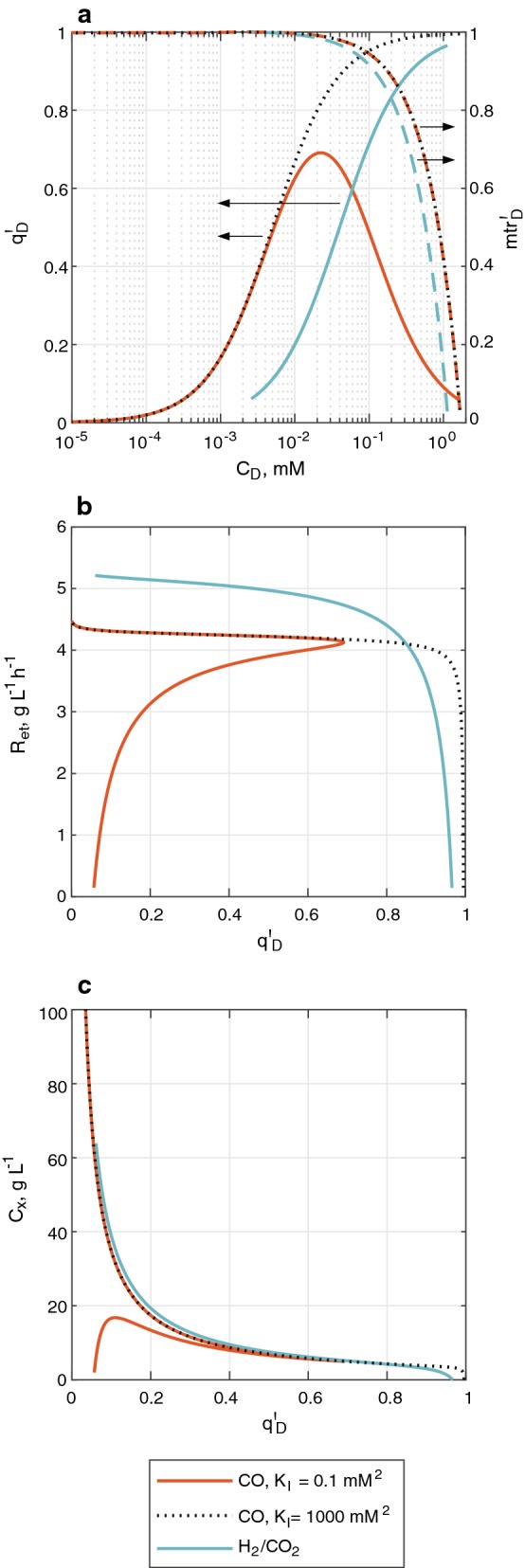
Relations between parameters used to describe bioreactor operational regimes for H_2_/CO_2_ and CO fermentations. **a** Dependency of non-dimensional electron donor uptake rate ($q^{\prime}_{D}$) and a non-dimensional mass transfer rate (${\text{mtr}}_{D}^{'} = \frac{{k_{L} a_{D} \cdot \left( {C_{D}^{*} - C_{D} } \right)}}{{{ \text{max} }\left[ {k_{L} a_{D} \cdot \left( {C_{D}^{*} - C_{D} } \right)} \right]}}$) on dissolved electron donor concentration ($C_{D}$); **b** relation between ethanol volumetric productivity ($R_{\text{et}}$) and $q^{\prime}_{D}$; **c** estimated biomass concentration ($C_{x}$) as function of $q^{\prime}_{D}$. The figure includes curves with black dotted lines that represent the operation of CO fermentation when the effect of substrate inhibition in the kinetic model is minimized by maximizing the value of $K_{I}$

The suboptimal regime is characterized by a low performance of the bioreactor and a $q^{\prime}_{D}$ approaching to 1 in H_2_/CO_2_ fermentation; due to the inhibition term used on the CO uptake kinetic equation, $q^{\prime}_{D}$ approaches to zero as CO concentration is highest (see Fig. 2a) resulting in a dual solution for $q^{\prime}_{D}$ as function of $C_{\text{CO}}$. According to the mass balances (see Additional file 1: Table S2), bioreactor productivity linearly depends on the mass transfer rate of the electron donors. Mass transfer rate concurrently depends on the mass transfer coefficient ($k_{L} a$) and the driving force (dissolved gas concentration gradient). $k_{L} a$ is determined by bioreactor design, the composition of the liquid phase, gas flow rate and gas sparging method [66], while the driving force is ruled by $C_{D}$ and the solubility of the gas components. As biomass concentrations ($C_{x}$) are low within the suboptimal regime (see Fig. 2a, c), $C_{D}$ is close to saturation. An elevated $C_{D}$ causes two unfavorable effects over bioreactor operation: (i) in the case of CO fermentation, it might inhibit CO consumption (see section “Gibbs free energy change of catabolic reactions”) mainly at the lower regions of the liquid column where the partial pressure is highest; and (ii) it limits the mass transfer driving force, which consequently hampers gas utilization and bioreactor productivity (see Fig. 2b). Yet, bioreactor performance improves sharply as $C_{x}$ increases and $q^{\prime}_{D}$ decreases to approximately 0.7, where mass transfer rate achieves 90% of its estimated maximum. This point marks the start of the optimal regime.

#### The ‘optimal’ operation regime

The optimal regime runs from a $q^{\prime}_{D}$ of 0.7 until it approaches zero. Within this range, mass transfer rate, ethanol volumetric productivity and gas utilization are above the 90% of their estimated maximum values (see Fig. 2a, b and Additional file 1: Figure S5). Since the value of $C_{x}$ (see Fig. 2c) is calculated by the optimization algorithm to linearize the term $q_{\text{CO}} \cdot C_{x}$ with respect to the mass transfer rate (see Additional file 1: Table S2), large increments on $C_{x}$ (or equivalently, large reductions on $C_{D}$ and $q^{\prime}_{D}$) would only return moderate improvements in bioreactor performance (see Fig. 2b). Therefore, working within the optimal regime would be desirable for continuous operation of the large-scale gas fermentor.

#### Influence of gas composition on bioreactor performance

This section compares bioreactor performance and the features of its operation between CO and H_2_/CO_2_ fermentations within the optimal regime. It may be assumed that a process fed by a gas with a composition falling in a determined point between the two composition boundaries (100% CO and 100% 3:1 H_2_:CO_2_ mixture), will behave proportionately to the contribution of each boundary.

##### $C_{{\text{et}}}$, $R_{\text{et}}$ and $U_{S}$

One parameter that showed to have a significant influence over bioreactor operation for CO and not for H_2_/CO_2_ fermentation is the liquid outflow rate ($\dot{V}_{L,o}$), which as well as $C_{x}$ is adjusted by the optimization algorithm to fulfill mass balances. $\dot{V}_{L,o}$ allows the removal of cells and the ethanol fraction that was not evaporated and transferred to the offgas. In CO fermentation, $\dot{V}_{L,o}$ is controlled by the biomass production rate for most of the $q^{\prime}_{D}$ range; this causes excessive ethanol removal along the liquid, preventing its accumulation within the bioreactor. Consequently, the ethanol concentration does not reach 45 g L^−1^ only until $q^{\prime}_{\text{CO}}$ is as low as 0.10 (where $C_{\text{CO}}$ is 1 × 10^−3^ mM and $C_{x}$ approximates to 20 g L^−1^) where biomass production rate lowers sufficiently; this could become another challenge for the fermentation process development since sustaining such low $q^{\prime}_{D}$ values can be difficult [32]. In H_2_/CO_2_ fermentation the relatively lower biomass production rates indirectly allow ethanol accumulation throughout the whole optimal regime due to the lower biomass yields (per mole of electron donor).

In order to lower the influence of $\dot{V}_{L,o}$ over bioreactor operation, biomass withdrawal may be decoupled from the removal of fermentation broth. Biomass retention within biofilms is known for increasing bioreactor productivity, yet in prolonged periods could lead to clogging [67]. If the biofilms were shaped into granules instead, clogging may be avoided and larger hydraulic loads can be handled by gas-lift bioreactors due to the high settling velocity of the granules [68]. Up to date there is no report on a gas fermentation set-up that uses biomass retention within granules, however a recent publication showed that *C. ljungdahlii* produces biofilms under stress by NaCl [69].

In general, the estimated biomass concentrations fermentation may seem unrealistic since the maximum $C_{x}$ reported for a continuous syngas fermentation using cell recycle is 10 g L^−1^ [46]. Although an explanation for this limitation has not been given in literature for gas fermentation, one hypothesis may be formulated based on the fact that the abiotic phase in a bioreactor undergoes spatial and temporal variations on the intensities of mixing and mass transfer [42]. If at one given moment inside one portion of the bubble column, the local value of $C_{\text{CO}}$ or $C_{{{\text{H}}_{ 2} }}$ was on the order of 0.01 mM, a 6% decrease in the local mass transfer coefficient (caused by the turbulent flow of the liquid phase) is enough to cause cells in a concentration of 10 g L^−1^ to lower that local $C_{D}$ by tenfold in approximately 0.03 s. Such variations may cause cells to temporarily (yet frequently) circulate through zones where the $C_{D}$ approaches to their thresholds, causing starvation. The detrimental effects of starvation on product generation and cells viability have been linked to the depletion of certain metabolic pools in fungi [40]. Considering the fact that depletion of the acetyl-CoA pool prevented *C. autoethanogenum* from achieving $C_{x}$ higher than 1.4 g L^−1^ in [32], it could be argued that the achievement of high values of $C_{x}$ in gas fermentations is not limited by the averaged rates of mass transfer, but instead to the slight spatial and temporal variations on those rates of mass transfer.

**Fig. 3:**
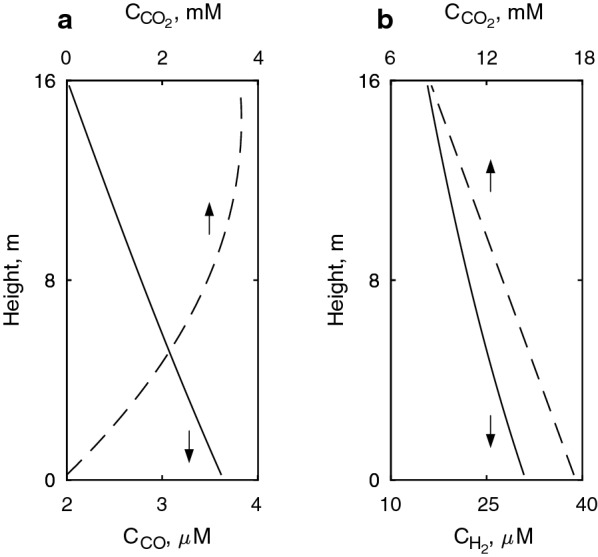
Gas concentration profiles along the liquid column for **a** CO fermentation and **b** H_2_/CO_2_ fermentation

Table 2 shows a summary of relevant parameters estimated within the optimal regime of CO and H_2_/CO_2_ fermentations; the values in the table describe the bioreactor operation at the liquid column height were the mean log pressure is found. The operation points shown were selected from the $q^{\prime}_{D}$ to satisfy the following conditions: (i) mass transfer is above the 90% of its maximum; (ii) CO does not inhibit its consumption at the bottom of the vessel (see Fig. 3a); (iii) the rate at which microbial biomass is being produced allows $C_{\text{et}}$ to reach 45 g L^−1^ in the liquid; (iv) H_2_ does not reach threshold concentrations at the top of the liquid column (see Fig. 3b), and (v) the concentration of biomass is not higher than 10 g L^−1^.

**Table 2:** Summary of relevant parameters of bioreactor operation and process performance for H_2_/CO_2_ and CO fermentations

Variable	Symbol	Unit	CO fermentation	H_2_/CO_2_ fermentation
Performance indicators				
Ethanol volumetric productivity	$R_{\text{et}}$	g L^−1^ h^−1^	4.25	5.1
Gas utilization	$U_{S}$	%	17.1	22.9
Gas outflow composition				
Hydrogen	$y_{{{\text{H}}_{2} }}$	mol mol^−1^	0.00	0.71
Carbon dioxide	$y_{{{\text{CO}}_{2} }}$	mol mol^−1^	0.11	0.24
Carbon monoxide	$y_{\text{CO}}$	mol mol^−1^	0.84	0.00
Ethanol	$y_{\text{et}}$	mol mol^−1^	0.01	0.01
Water	$y_{w}$	mol mol^−1^	0.04	0.04
Concentrations in the fermentation broth^a,b^				
Hydrogen	$C_{{{\text{H}}_{ 2} }}$ ($C_{{{\text{H}}_{ 2} }}^{*}$)	mol m^−3^	0.00 (0.00)	0.025 {0.033; 0.018} (1.15 {1.63; 0.78})
Carbon dioxide	$C_{{{\text{CO}}_{ 2} }}$ ($C_{{{\text{CO}}_{2} }}^{*}$)	mol m^−3^	0.32 {0.00; 4.22}	12.46 {17.11; 8.94} (13.09 {18.51; 8.86})
Carbon monoxide	$C_{\text{CO}}$ ($C_{\text{CO}}^{ *}$)	mol m^−3^	2.7 × 10^−3^ {3.6 × 10^−3;^ 2.0 × 10^−3^} (1.62 {2.45; 1.01})	0.000 (0.000)
Ethanol	$C_{et}$	mol L^−1^ (g L^−1^)	0.96 (44.3)	0.98 (45.0)
Biomass	$C_{x}$	Cmol m^−3^ (g L^−1^)	395 (10.0)	399 (10.1)
Parameters estimated with thermodynamics^a^				
Catabolic energy production	$\Delta G_{\text{cat}}^{{0^{\prime}}}$	kJ mol_CS_^−1^	− 29.2 {− 48.2; − 24.0}	− 19.9 {− 23.0 − 16.45}
Biomass yield	$Y_{x/CS}$	Cmol_*x*_ mol_CS_^−1^	0.041	0.020
Biomass specific consumption/production rates (logarithmic mean)				
Hydrogen	$q_{{{\text{H}}_{ 2} }}$	mol Cmol_*x*_^−1^ h^−1^	0.00	− 1.67
Carbon dioxide	$q_{{{\text{CO}}_{2} }}$	mol Cmol_*x*_^−1^ h^−1^	1.00	− 0.56
Carbon monoxide	$q_{\text{CO}}$	mol Cmol_*x*_^−1^ h^−1^	− 1.52	0.00
Ethanol	$q_{\text{et}}$	mol Cmol_*x*_^−1^ h^−1^	0.23	0.28
Water	$q_{w}$	mol Cmol_*x*_^−1^ h^−1^	− 0.73	0.84
Cells	$\mu$	h^−1^	0.06	0.01
Non-dimensional electron donor uptake rate	$q^{\prime}_{D}$	–	0.35	0.38
Streams entering and leaving the bioreactor				
Gas flow rate at the top	$F_{G,t}$ ($\dot{V}_{G,t}$)	mol s^−1^ (m^3^ s^−1^)	462 (7.8)	418 (7.1)
Gas flow rate at the bottom	$F_{G,b}$ ($\dot{V}_{G,b}$)	mol s^−1^ (m^3^ s^−1^)	479 (4.0)	528 (4.4)
Liquid outflow rate	$\dot{V}_{L,o}$	m^3^ h^−1^	30.0	39.3
Fresh syngas	$F_{S}$	mol s^−1^	80.0	118
Parameters regarding gas and liquid flows and mixing (logarithmic mean)				
Gas flow rate	$F_{G }$($\dot{V}_{G}$)	mol s^−1^ (m^3^ s^−1^)	471 (5.6)	471 (5.6)
Superficial gas velocity (pressure-corrected)	$v_{sG}^{c}$	m s^−1^	0.14	0.14
Liquid flow rate	$\dot{V}_{L}$	m^3^ s^−1^	26.9	26.9
Mixing time	$t_{m}$	s	60.4^c^ 54.3^d^	61.2^c^ 54.3^d^
Mass transfer coefficients (logarithmic mean)				
Hydrogen	$k_{L} a_{{{\text{H}}_{2} }}$	s^−1^	0.000	0.164
Carbon dioxide	$k_{L} a_{{{\text{CO}}_{ 2} }}$	s^−1^	0.000	0.098
Carbon monoxide	$k_{L} a_{\text{CO}}$	s^−1^	0.104	0.000

^a^The average value is shown first, followed by the values at the top and the bottom of the liquid column between curly brackets

^b^The values between round brackets represent the saturation concentrations of CO, H_2_ and CO_2_

^c^Simulated using the 9 vertically stacked compartments model

^d^Calculated with Eq. (21)

In such points of operation, H_2_/CO_2_ fermentation returns a 19% higher ethanol productivity (5.1 g L^−1^ h^−1^) than fermentation of CO (4.3 g L^−1^ h^−1^); this difference is attributed to a higher mass transfer rate in H_2_/CO_2_ fermentation mainly because the higher H_2_ diffusivity in water (see section “Thermodynamics-based black-box model of microbial reactions“) makes $k_{L}$ in $k_{L} a$ 58% higher for H_2_ compared to CO (see Table 2). Considering that ethanol productivities have reportedly reached 8 g L^−1^ h^−1^ through fermentation of CO-rich syngas [70], and that commercial sugar-based fermentations commonly fall between 1.5 and 2.0 g L^−1^ h^−1^ [71], it can be argued the estimations made in this study do not fall out of context and even more important, they could be subjected to further improvement.

Moreover, the higher mass transfer rates in H_2_ have a similar effect on gas utilization ($U_{s}$, see section “Thermodynamics-based black-box model of microbial reactions”) in relation to CO fermentation. However, $U_{s}$ does only reach 23% in absolute terms, which makes the gas recycling step (see section “Process configuration”) a necessity to guarantee full use of the fresh gas fed to the process. Therefore, an upstream operation for gas composition control is essential to avoid the accumulation of gases within the gas recycle.

Lastly, the CO, H_2_ and CO_2_ mass transfer coefficients used in the present work range between 0.05–0.21, 0.08–0.33 and 0.05–0.20 s^−1^, respectively. Unfortunately, mass transfer coefficients for the transfer of CO, H_2_ and CO_2_ are available only for laboratory-scale bioreactors [72–74] where the heterogeneous bubbling regime may not be achieved [66] and therefore, the predicted values cannot be compared with reported experiments. However, the estimated ranges agree well with the experimental data (corrected with the gas diffusivities in water, see section “Mass transfer-based model of the industrial bioreactor”) reported for oxygen transfer within large bubble columns by [75].

##### Energy requirements

**Fig. 4:**
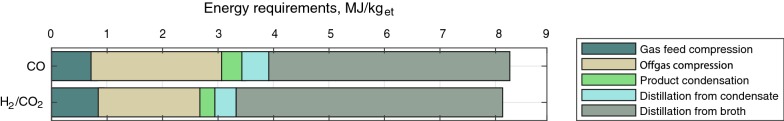
Breakdown of energy requirements of the proposed process configuration

When CO is fed to the reactor, roughly 60% of its carbon goes to CO_2_. This causes the molar gas flow rate across the reactor to slightly decrease (see Table 2). Contrarily, when the 3:1 H_2_:CO_2_ mixture is fed to the fermentor, the two gases are consumed and none is produced; thus the molar gas flow diminishes by 20%. This difference in gas flow profiles impacts significantly on the two largest contributors to total energy requirements, i.e., compression of the gas streams (in agreement with [21]) and product distillation (see Fig. 4).

**Fig. 5:**
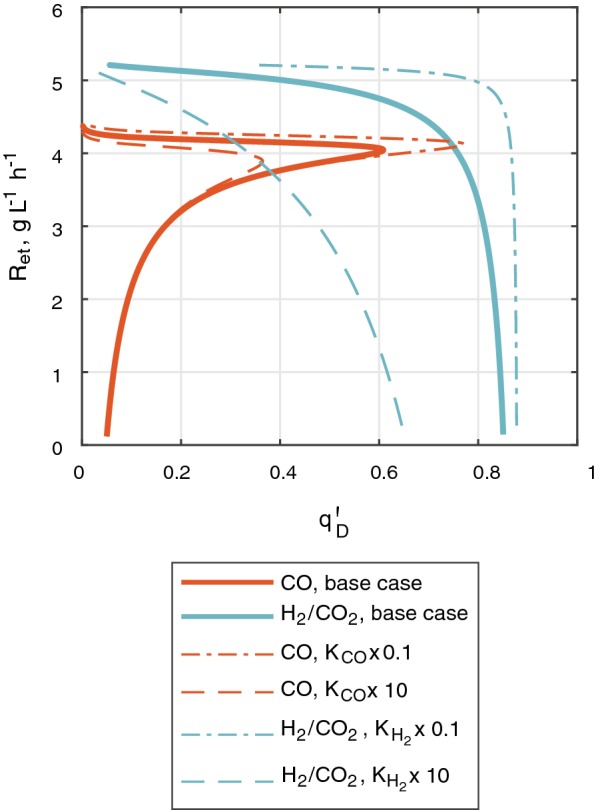
Influence of half-saturation constants on ethanol productivity in the large-scale gas fermentor

In H_2_/CO_2_ fermentation less power is needed to compress the recycling offgas compared to CO fermentation. Furthermore, as the productivity in H_2_/CO_2_ fermentation is higher and the offgas’ ethanol evaporation capacity lower (due to lower offgas flow rate), the liquid outflow in the chosen point of operation (see Table 4) is larger than in CO fermentation. As consequence, the distillation of the diluted broth consumes more energy in the H_2_/CO_2_ fermentation. All in all, the total absolute energy requirements are higher for the H_2_/CO_2_ fermentation; however due to the higher ethanol productivity, the energy needed per unit of ethanol produced is lower than in CO fermentation (see Fig. 5).

### Sensitivity analysis

**Fig. 6:**
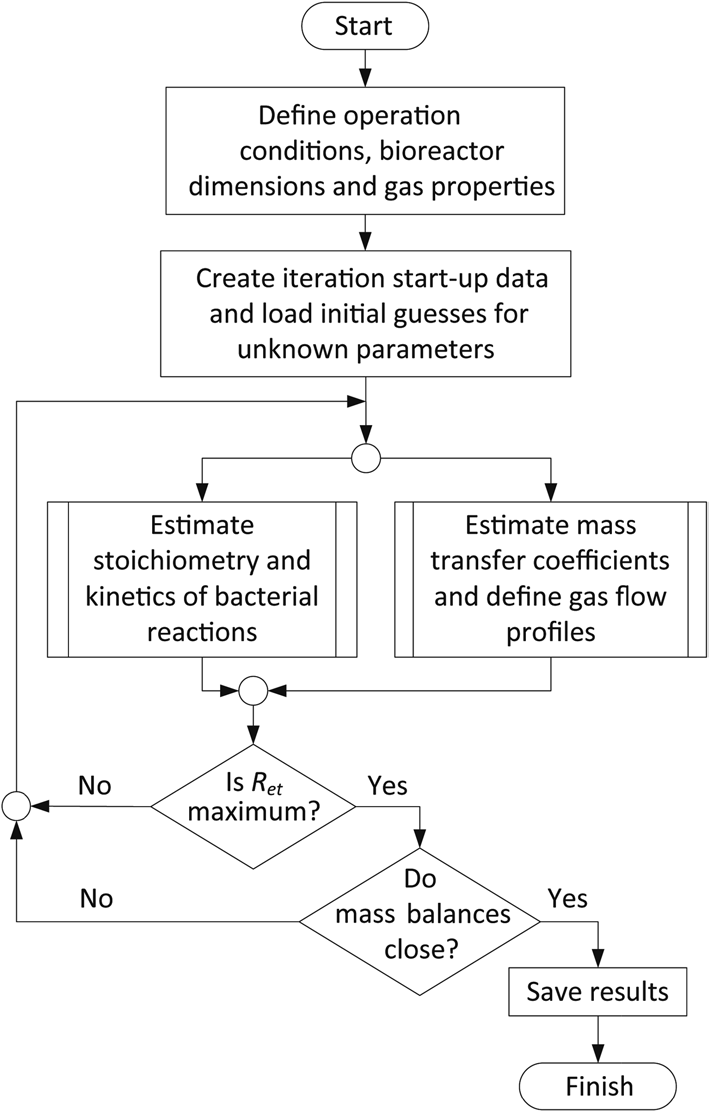
General structure of the calculation process for optimizing productivity. The figure is based on [88]

The sensitivity analysis assesses the impact of the value that half-saturation constants and maximum substrate uptake rate would apply on bioreactor performance. If $K_{\text{CO}}$ and $K_{{{\text{H}}_{ 2} }}$ decreased by tenfold, the appearance of the optimal regime of operation would occur at higher $q^{\prime}_{D}$ (see Fig. 6), which in an industrial setting could improve bioreactor operation robustness to withstand fluctuations on $C_{x}$ and $k_{L} a$. In the opposite case, if $K_{\text{CO}}$ and $K_{{{\text{H}}_{ 2} }}$ were 10 times higher than what was fixed in section “CO and H_2_ half-saturation constants”, the preservation of a stable optimal regime could require delicate control of low $q^{\prime}_{D}$. This last case could severely affect H_2_/CO_2_ fermentation as $C_{{{\text{H}}_{ 2} }}$ needs to be kept at relatively higher values (see Table 2) to avoid reaching a threshold concentration at the top of the fermentor. Therefore, $K_{{{\text{H}}_{ 2} }}$ as well as $K_{\text{CO}}$ need to be in the order of 1 × 10^−2^ mM or lower.

When the maximum uptake rate is decreased by 60% from the value estimated with Eq. (7), a negligible effect is seen on bioreactor productivity and gas utilization within the optimal regime of bioreactor operation.

Further, as the relation between the rates of mass transfer and consumption of the electron donors is highly linear (see mass balances in Additional file 1: Table S2), a 100% improvement on the mass transfer coefficients (with respect to the values shown in Table 2) would result in an 86% improvement on bioreactor productivity. That means that ethanol productivity for CO and H_2_/CO_2_ fermentations could be as high as 7.9 and 9.4 g L^−1^ h^−1^, respectively. Similarly, due to the improvement on gas transfer to the liquid, gas utilization would rise to 31 and 38% for CO and H_2_/CO_2_ fermentations, respectively. If contrarily, mass transfer coefficients were 50% smaller than predicted for gas transfer to pure water (see section “Mass transfer-based model of the industrial bioreactor”) both ethanol productivity and gas consumption would roughly be cut by half.

The effect of an increased microbial tolerance to ethanol such that $C_{et}$ may be maintained at 80 g L^−1^, is only reflected on the energy requirements which in the case of H_2_/CO_2_ fermentation would decrease by 30% to 5.8 MJ kg_et_^−1^. A similar decrease in energy requirements would be seen for the fermentation of CO a as long as the concentration of biomass climbed to 25 g L^−1^ at a $q^{\prime}_{D}$ of 0.13, where $\mu$ is low enough to allow ethanol concentration to rise from 45 g L^−1^.

Moreover, if the aspect ratio was increased to 10 while maintaining the volume at 700 m^3^ (vessel height and diameter will be 44.7 and 4.5 m, respectively), the gas utilization will climb to 35 and 44% for CO and H_2_/CO_2_ fermentations, respectively, mainly due to the increased gas retention times. As a consequence of the increased gas utilization, the ethanol productivity may slightly rise to 5.3 and 6.7 g L^−1^ h^−1^ for the same two cases as previously. Finally, since the hydrostatic pressure at the bottom of the bioreactor will increase with the height of the liquid column, the total power requirements will consequently rise by an average of 11% for the two gas compositions because increase need for gas compression. Furthermore, since the relation between the gas hold-up and the productivity is also highly linear, a 100% increase in the hold-up to 0.30 m_*G*_^3^ m_*G+L*_^−3^ will be reflected as an equivalent increase on ethanol productivity to 8.6 and 9.4 g L^−1^ h^−1^ for CO and H_2_/CO_2_ fermentations, respectively. However, due to the reduced gas retention time, the gas utilization will fall to 11 and 14% for the two fermentation cases. Therefore, a balance may have to be established between the height of the bioreactor and the gas hold-up to guarantee that productivity rises without significantly affecting the gas consumption or the energetic demands of the overall process.

Finally, if the gases provided to the fermentation were not pure, as it is likely in an industrial setting, the gas recycling will not be possible. Such process configuration may cause the energy requirements to rise by 15% to 9.6 and 9.5 MJ kg_et_^−1^ for CO and H_2_/CO_2_ fermentations, respectively. The reason behind this result is the fact that the energy savings on the offgas compression are not sufficiently high to counter the extra expenses derived from the compression of higher amounts of gas at the bioreactor inlet. Industrial sources of CO, H_2_ and CO_2_, such as syngas and steel manufacturing offgases, which contain impurities that if recycled may accumulate inside the bioreactor, may have to deal with the extra energy expenses of not using gas recycle. On the other hand, the fermentation of mixtures between H_2_ produced for example, through the electrolysis of water, and CO_2_ recovered from a generic combustion process may be benefited by the energetic advantage of using gas recycling.

## Conclusions

An alternative model for simulating gas fermentation within a large-scale bubble column bioreactor was developed. The model coupled a thermodynamics-based black-box model of main microbial reactions with a mass transfer-based model of the bioreactor. A significant amount of effort was put on validating the black-box model predictions with trends and data found in literature for acetogens or microorganisms using similar catabolic processes:

- The estimated threshold concentrations for CO, H_2_ and CO_2_ agreed with reported data for acetogens during early and late growth stages at different temperatures.
- Predicted biomass specific uptake rates for CO and H_2_ consumption surpassed reported values by 250% suggesting that there might exist a potential for strain improvement.
- Estimation of substrate half-saturation constants form threshold concentrations proved to yield results comparable with data reported for CO and H_2_ consumption by acetogenic and methanogenic microorganisms.

The large-scale gas fermentor simulation showed that ethanol productivities may reach between 4.3 and 5.1 g L^−1^ h^−1^ and CO and H_2_ utilization per step may not surpass 23%. If instead, mass transfer coefficients were 100% higher than the estimated by the model developed for oxygen transfer to pure water, then productivities may achieve 7.9 and 9.4 g L^−1^ h^−1^ while gas utilization may climb to 38%. Such performance indicators are obtained if H_2_ does not achieve threshold concentrations at the top of the liquid column, CO consumption is not inhibited at the bottom of the bioreactor, ethanol concentration reaches 45 g L^−1^ and if biomass withdrawal from the bioreactor was decoupled from the fermentation broth removal.

It is recommended that multi-objective optimizations are done to further validate the bioreactor performance results by comparing them with reported data and process configurations that have been proposed and patented. The model could also be used to acquire a broader view on how process performance, especially gas utilization, could be further improved. In addition, the black-box model may be extended to include intracellular processes relevant for energy conservation and guide further understanding on the factors influencing the selectivity between ethanol and acetic acid in acetogens.

## Methods

This section describes the structure of the hybrid model, the estimation of relevant parameters and how process performance is assessed.

### The hybrid model

A deterministic model for simulating ethanol production in a large-scale gas fermentor is proposed. The model consists of two main parts: (i) a thermodynamics-based black-box model of the microbial reactions and (ii) a model of the fermentor hydrodynamics. Two gas compositions are evaluated separately to represent the two boundaries of possible compositions that acetogenic bacteria are able to catabolize, i.e, pure CO and a 3:1 mixture of H_2_ and CO_2_, which, respectively, may be obtained industrially from the offgas of steel manufacturing [70] and by mixing the CO_2_ recovered from a generic combustion process with H_2_ produced from, for instance, the electrolysis of water [76].

The simulation of the large gas fermentor is here done by assuming that a generic acetogenic bacterial strain has been adapted, modified or that the conditions in the bioreactor are such that the net rate of acetic acid production is zero, although it is commonly reported that the main product of acetogens is acetic acid while ethanol is generally a co-product. The assumption is sustained on the fact that ethanol production form CO and H_2_ as electron donors is thermodynamically feasible on its own, disregarding the current understanding of the physiology of acetogens and the limitations of gene editing techniques applied to these bacteria.

#### Thermodynamics-based black-box model of microbial reactions

The microbial metabolism is considered to be formed by catabolism and anabolism. Ethanol is a product of CO or H_2_/CO_2_ catabolism, while cells are the product of anabolism starting from the same energy and carbon sources; CO and H_2_ are the energy sources or electron donors ($D$), while CO and CO_2_ are the carbon sources (CS). Table 1 shows the stoichiometry of catabolism (Eqs. 1–4), whereas Eqs. (10) and (11) show the stoichiometries of anabolism. If thermodynamically feasible ($\Delta G^{{0^{\prime}}} < 0$), the amount of Gibbs free energy released by catabolism is mainly used to support cell growth. Biomass yields ($Y_{x/CS}$ in Eq. 6) are then calculated from the ratio between Gibbs free energy dissipation during growth ($\Delta G_{\text{dis}}$ in Eq. (12), where $c$ is the number of carbon atoms in the CS and $\gamma$ is its degree of reduction—electrons available for redox exchange) and free energy change in catabolism ($\Delta G_{r}^{{0^{\prime}}}$ in Eq. 5) plus the ratio between the degrees of reduction of biomass material and of $D$ [77]. In Eq. (6), the term $\left( {\Delta G_{\text{dis}} /\Delta G_{\text{cat}}^{{0^{\prime}}} } \right)$ represents the amount of CS needed to produce the necessary amount of free energy to produce 1 Cmol of biomass ($x$); the term $\left( { \gamma_{x} /\gamma_{D} } \right)$ represents the amount of CS required for stoichiometrically building 1 Cmol_*x*_. Finally, the term $\nu_{D}^{\text{an}}$ (stoichiometric coefficient of $D$ in anabolism) in Eq. (6), is used since $D$ is not the CS in H_2_ catabolism. Moreover, the stoichiometric coefficient of any $j$th component in the metabolic reactions (mol_j_ Cmol_*x*_^−1^) is determined by adding the contributions of the catabolic and anabolic reactions (see Eq. 13).

10 $$- 2{\text{CO}} - \frac{1}{4}{\text{NH}}_{4}^{ + } - \frac{1}{2}{\text{H}}_{2} {\text{O}} + {\text{CH}}_{1.75} {\text{O}}_{0.5} {\text{N}}_{0.25} + {\text{CO}}_{2} + \frac{1}{4}{\text{H}}^{ + }$$

11 $$- 2{\text{H}}_{2} - {\text{CO}}_{2} - \frac{1}{4}{\text{NH}}_{4}^{ + } + {\text{CH}}_{1.75} {\text{O}}_{0.5} {\text{N}}_{0.25} + \frac{3}{2}{\text{H}}_{2} {\text{O}} + \frac{1}{4}{\text{H}}^{ + }$$

12 $$\Delta G_{\text{dis}} = 200 + 18 \cdot \left( {6 - c} \right)^{1.8} + { \exp }\left\{ {\left[ {\left( {3.8 - \frac{{\gamma_{CS} }}{c}} \right)^{2} } \right]^{0.16} \cdot \left( {3.6 + 0.4c} \right)} \right\}$$

13 $$\nu_{j}^{\text{met}} = \nu_{j}^{\text{cat}} \left( {\frac{{\Delta G_{\text{dis}} }}{{\Delta G_{\text{cat}}^{{0^{\prime}}} }}} \right) + \nu_{j}^{\text{an}} \left( {\frac{{\gamma_{x} }}{{\gamma_{D} \cdot \nu_{D}^{\text{an}} }}} \right)$$

Since the Gibbs free energy change ($\Delta G_{r}^{{0^{\prime}}}$ in Eq. 5) is calculated at physiological conditions, its magnitude and the parameters derived from it (e.g, biomass yield and stoichiometry of metabolic reactions) will depend on temperature and the activity of the *m* products and substrates at the intracellular space. It has been argued that “the choice of the species used for calculation of $\Delta G_{r}^{{0^{\prime}}}$ of a reaction $r$ should be based on the species for which the activity is closest to the reference activity (i.e, 1 mol L^−1^ for aqueous species, 1 atm for gases)” [43]. Therefore, the value of $C_{j}$ in Eq. (7) corresponds to the aqueous concentrations for: ethanol, NH_4_^+^ and H^+^ ions, while for the gases: CO, H_2_ and CO_2_, their partial pressures are used.

Water intervenes as product and reactant in the catabolic reactions (see Eqs. 1–4). However, the concentration of electron donors, ethanol, CO_2_ and H^+^ ions are generally very low and catabolic reactions take place within a “dilute aqueous system” [77]. Therefore, the concentration of water is not considered in the calculation of $\Delta G_{r}^{{0^{\prime}}}$ with Eq. (5) [49, 77].

As CO, H_2_ and CO_2_ are uncharged gases, they can freely diffuse across the cell membrane and thus their concentrations are assumed to be the same inside and outside the cells. The same is assumed for ethanol whose concentration ($C_{\text{et}}$) is used as a fixed value at 45 g L^−1^, which approximates to the highest concentration achieved in a syngas fermentation [78].

The concentration of the dissolved gases is assumed to vary within a large range since, as it will be explained in section “Interlink between both models”, they are the link between the model of microbial reactions and the mass transfer model and largely influence the bioreactor performance. Table 3 summarizes the values of intracellular concentrations of the reactants and products of catabolic reactions (Eqs. 1–4).

**Table 3:** Intracellular concentrations of substance involved in catabolic reactions

Substance	Concentration, mol L^−1^
Fixed values	
H^+^ ions	1.0 × 10^−7^ [64]
NH_4_^+^ ions	1.0 × 10^−1^ [79]^a^
Ethanol	9.8 × 10^−1^
Ranges of values^b^	
CO	1 × 10^−8^–1 × 10^−3^
H_2_	1 × 10^−8^–1 × 10^−3^
CO_2_	1 × 10^−6^–1 × 10^−1^

^a^Defined from a 0.1 M ionic strength

^b^Ranges of dissolved gas concentrations were defined based on the corresponding range of partial pressures between 1 × 10^−5^ to 1 atm

The effect of ionic strength is neglected from the calculation of Gibbs free energy changes since an ionic strength as high as 0.1 M would result in variations of maximum 0.6 kJ mol^−1^ for the reactions shown in Table 1 [79]. Therefore, the activity coefficients of all substrates involved in the considered microbial reactions are rounded to 1.

The reaction rates of microbial metabolism are calculated by linearly linking their stoichiometry to the hyperbolic substrate uptake kinetics of the electron donors (i.e, CO and H_2_—see Eqs. 14 and 15). Such kinetic relations were reported to be applicable to CO consumption by *C. ljungdahlii* [47] and H_2_ consumption by *C. ragsdalei P11* [50]. In Eqs. (14) and (15), $K_{\text{CO}}$ and $K_{{{\text{H}}_{ 2} }}$ are the half-saturation constants while $K_{I}$ is the inhibition constant for CO (0.1 mol^2^ m^−6^ [47]); section “Model validation” describes the procedure followed to assess and set the values for the half-saturation constants. The maximum substrate uptake rate ($q_{D}^{ \text{max} }$) is calculated for CO and H_2_ from the theoretical maximum rate of electron consumption by cells, as shown in Eq. (7) [49]. $q_{D}^{ \text{max} }$ is a function of the temperature and the degree of reduction of the electron donor. Equation (7) was formulated based on a “maximum rate of electron transport in the catabolic energy production” of 3 mol_*e*_− Cmol_*x*_^−1^ h^−1^ at 20 °C and which was found to fit uptake data from *E. coli* growing on different substrates [49].

14 $$q_{\text{CO}} = q_{\text{CO}}^{ \text{max} } \cdot \frac{{C_{\text{CO}} }}{{K_{\text{CO}} + C_{\text{CO}} + \frac{{C_{\text{CO}}^{2} }}{{K_{I} }}}}$$

15 $$q_{{{\text{H}}_{ 2} }} = q_{{{\text{H}}_{ 2} }}^{ \text{max} } \cdot \frac{{C_{{{\text{H}}_{ 2} }} }}{{K_{{{\text{H}}_{ 2} }} + C_{{{\text{H}}_{ 2} }} }}$$

#### Model validation

The thermodynamics-based black-box model of microbial reactions is validated by comparing its results against general tendencies observed in reported experiments and data. The estimations of the Gibbs free energy change of catabolic reactions is compared to published experimental data in terms of threshold concentrations of the gases, i.e., the concentrations at which the catabolic reaction returns a minimum amount of energy necessary to power the proton motive force (− 15 kJ mol_H+_^−1^ [77], although it could be as low as − 9.1 kJ mol_H+_^−1^ in acetogens [80]).

In addition, it has been suggested that the value of the substrate half-saturation constants are likely close to the threshold concentrations of that substance; further in poorly soluble substances, the same constants can also be expected to approximate to their solubility in the aqueous phase [77]. Thus, the values of the $K_{\text{CO}}$ and $K_{{{\text{H}}_{ 2} }}$ found in literature are first judged against those two criteria and when necessary, modified to a value in accordance with the restrictions.

Lastly, the estimated values for maximum substrate uptake rates and biomass yields are also compared with published data for acetogens or microorganisms that use similar catabolic routes.

#### Mass transfer-based model of the industrial bioreactor

The height and volume of the industrial bubble column bioreactor are both fixed at 20 m and 700 m^3^, respectively. The stated height is common in industry [81] while the volume is relatively large yet regarded as cost efficient [82]. Considering that the reactor will have a 20% overhead space and a gas hold-up fixed at 15% [83], the fermentation broth (liquid phase) will occupy 476 m^3^. More details of the model parameters for the bubble column are shown in the Table 4. In addition, since the aspect ratio and the gas hold-up are parameters which can adopt different values in industry, the effect of different values that those shown in Table 4, is assessed within the sensitivity analysis.

**Table 4:** Model parameters for bubble column bioreactor design and operation during gas fermentation

Parameter	Unit	Value
Operation conditions		
Temperature	K	310.15
Top pressure	Pa	1.52 × 10^5^
Gas hold-up	m_*G*_^3^ m_*G+L*_^−3^	0.15
pH	–	5.0
Maximum ethanol concentration^a^	mol m^−3^	1304
Bioreactor dimensions		
Volume	m^3^	700
Height	m	20
Aspect ratio	–	3.0
Diameter	m	6.7
Overhead space	%	20
Height of gas–liquid mixture	m	16
Relevant gas properties for the mass transfer model (at 37 °C)		
Diffusivities^b^		
O_2_ CO H_2_ CO_2_	m^2^ s^−1^	3.21 × 10^−9^ 2.88 × 10^−9^ 4.55 × 10^−9^ 2.70 × 10^−9^
Henry’s coefficient^c^		
CO H_2_ CO_2_	mol_S_ m^−3^ Pa^−1^	0.79 × 10^−5^ 0.72 × 10^−5^ 24.6 × 10^−5^

^a^Liquid–vapor equilibria data for the ethanol/water system were estimated using the non-random two-liquid model for calculating activity coefficients (see Additional file 1: Table S3)

^b^Estimated according to the method presented by Wilke and Chang [84]

^c^Estimated according to the method presented by Sander [85]

The mass transfer model is defined considering the coexistence of two phases inside the bioreactor: (i) a liquid phase which initially is assumed to have a homogeneous composition, and (ii) a gaseous phase which behaves as a plug flow. Although bacterial cells would constitute a third phase within the bioreactor, they are considered to occupy a negligible volume and to be homogeneously distributed within the liquid phase; thus biomass would not influence mass transfer. As a first approach, the fermentation broth is assumed to be a coalescing liquid despite the fact that ethanol inhibits water coalescence depending on the alcohol concentration [86]. For simplicity, the liquid dynamically behaves as pure water under a heterogeneous bubbling regime inside the bubble column [66]; bubbles are thus assumed as coarse with a 6 mm average diameter [66]; with these assumptions, the volumetric mass transfer coefficients ($k_{L} a_{S}$) and the gas hold-up ($\varepsilon_{G}$) are estimated from the pressure-corrected superficial gas velocity ($v_{Gs}^{c}$) using Eqs. (16) and (17) [66, 83]; such equations have been derived by fitting experimental data obtained in bubble columns with a diameter and height between 0.08–11.6 m and 0.3–21 m, respectively [66]. $v_{Gs}^{c}$ is estimated from the logarithmic mean of the volumetric gas flow rates across the bioreactor calculated at normal conditions of temperature and pressure (see Additional file 1: Table S3 for the specific equations used for the calculation). The gas flow rates and the estimated $v_{Gs}^{c}$ are ultimately constrained to return a $\varepsilon_{G}$ fixed at 0.15. Although the gas hold-up is not a good design closing criteria, it was chosen to limit further maximization of mass transfer coefficients and subsequent minimization of gas use (see section “Technical performance”).

16 $$k_{L} a_{S} = \left( {0.32 \cdot v_{Gs}^{c0.7} } \right) \cdot \left[ {1.022^{{\left( {T - 293.15} \right)}} } \right]\left( {\frac{{{\mathcal{D}}_{S} }}{{{\mathcal{D}}_{{O_{2} }} }}} \right)$$

17 $$\varepsilon_{G} = 0.6 \cdot v_{Gs}^{c0.7}$$

Mass balances are established around the gas fermentor for all species involved in the metabolic reactions leading to ethanol and bacterial biomass production (see Additional file 1: Table S2). Furthermore in the energy balances, the power requirement for compression of the gas feed and the offgas for gas recycle is estimated assuming adiabatic operation of compressors with a mechanical efficiency of 0.7 [28]. The heat required for gas cooling is obtained using average heat capacities within the applied temperature ranges, assuming that condensation will occur only when the final cooling temperature has been reached; a refrigeration coefficient of performance of 3.7 is assumed [87]. Additional file 1: Table S4 shows the specific equations used for estimating energy requirements.

#### Interlink between both models

The thermodynamics-based model of microbial reactions as well as the mass transfer-based model of the bioreactor converge in the concentration of the dissolved gases and ethanol in the liquid phase. The dissolved concentration of the electron donor ($C_{D}$ where $D$ is either, CO or H_2_) is fixed before solving the mass balances to avoid unnecessary issues with convergence into a solution. Thus, a range of steady-state operation points of the gas fermentor are estimated for a range of values of $C_{D}$. Although the system has no degrees of freedom (see the list of decision variables below and equations SI1 to SI8 in the Additional file), it does have multiple solutions; therefore, an optimization is used to obtain a solution that maximizes the ethanol volumetric productivity ($R_{\text{et}}$ in Eq. 18) while minimizing power consumption.

18 $$R_{\text{et}} = q_{\text{et}} \cdot C_{x}$$

The optimization uses the fraction of ethanol that exits the bioreactor along the liquid phase as objective function; whereas, the biomass concentration, the molar gas inflow and outflow rates, the CO, H_2_ and CO_2_ contents in the offgas, the concentration of ethanol and CO_2_ (only in the case of H_2_/CO_2_ fermentation) in the liquid phase and the liquid outflow rate from the bioreactor are the decision variables. The optimization is executed by a sequential quadratic programming (‘sqp’) algorithm implemented in the ‘fmincon’ function in MatLab R2017b. The system of mass balance equations outlaid for the bioreactor model and thermodynamic feasibility of the catabolic reactions are used as constraints within the optimization. The calculation process is schematized in the Fig. 6.

### Gas concentration profiles

It is important to assure that at a determined operation point, the microbial uptake of electron donors does not meet gas concentrations that lead to either unfeasible catabolic reactions at the top of the liquid column or inhibition at the bottom (in the case of CO). Thus the dissolved CO, CO_2_ and H_2_ concentration profiles along the height of the liquid column are estimated by linearly discretizing the *y*-axis into 9 ideally mixed compartments stacked vertically. The initial assumption of the liquid phase having a homogenous composition is corrected by this calculation.

The approach is similar to that used in [89], where the height of the liquid column ($H_{\text{mix}}$) has three mixing cells stacked vertically (see Eq. 19 [89]); then each cell is subdivided into three compartments stacked horizontally which recreate the effect of dispersion on the radial direction assuming the liquid flow will follow a helicoidal stream line.

19 $$n_{c} = 0.8 \cdot \frac{{H_{\text{mix}} }}{\tau }$$

Mass balance equations are constructed in each compartment assuming the liquid phase will be exchanged between the adjacent segments at a flow rate determined by Eq. (20) [66]; biomass concentration is assumed homogeneously distributed within the whole liquid volume. The gas phase is assumed to behave as a plug flow. Mass transfer properties in each segment are found using the same methodology as explained in section “Mass transfer-based model of the industrial bioreactor” since it is assumed that each compartment would behave as a shallow bubble column.

20 $$\dot{V}_{L} = 0.3 \cdot \tau^{{\frac{5}{3}}} \cdot \left( {g \cdot \dot{V}_{G} } \right)^{{\frac{1}{3}}}$$

The compartmentalization scheme is validated by calculating the mixing time using two approaches, i.e., (i) using Eq. (21) [89] and, (ii) simulating a mixing time-determination experiment, in which a tracer is injected at the top compartment; the mixing time is defined as the time it takes for the tracer concentration at the top compartment to reach 95% of the final concentration.

21 $$t_{m} = \frac{{N_{\text{mix}} \cdot \tau^{{\frac{2}{3}}} }}{{\left( {g \cdot v_{Gs}^{c} } \right)^{{\frac{1}{3}}} }}$$

### Process configuration

**Fig. 7:**
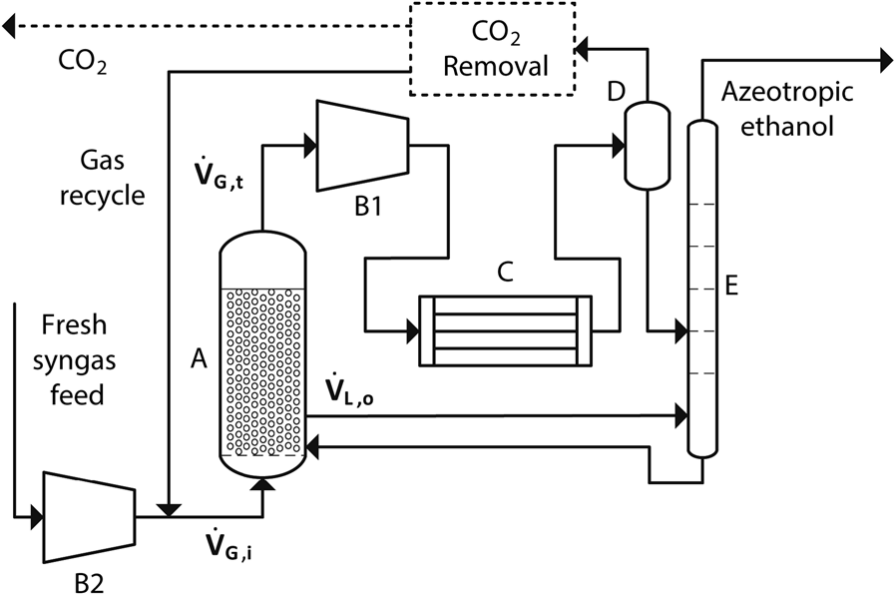
Conceptual process configuration; A: bubble column bioreactor, B1 and B2: gas compression, C: cooling and condensation, D: flash separation, E: azeotropic distillation. For the case in which the H_2_/CO_2_ mixture is fed into the bioreactor, the CO_2_ removal unit is not needed

Fresh gas is first mixed with a stream of recycled offgas and is then fed to the large-scale bioreactor. The fermentable gas is consumed during fermentation and ethanol is produced. At the exit, the offgas is compressed to 3.1 × 10^5^ Pa (a pressure equal to the bioreactor bottom pressure—see Table 4) and cooled to − 6 °C in order to condense nearly 100% of the water–ethanol mixture. This condensate stream along with the fermentation broth is sent to distillation. Figure 7 shows the conceptualized process configuration.

When pure CO is fed to the fermentation, the dry offgas undergoes CO_2_ removal prior to recycling; this operation is not needed if H_2_/CO_2_ was used as feedstock. Since gas recycling has not been included in process designs reported in literature [90], the possible effects of not including it are discussed within the sensitivity analysis.

### Process assessment

#### Technical performance

In addition to $R_{\text{et}}$, the process technical performance is evaluated from the perspective of the gas utilization ‘per step’ ($U_{S}$) inside the bioreactor (Eq. 22) and the energy requirements of the fermentation plus the ethanol separation processes. $U_{S}$ is calculated as the ratio between the amounts of $D$ depleted across the reactor and the $D$ fed (fresh plus recycled). Energy requirements account for: (i) power for compression of the gas feed and the offgas, (ii) power for condensation of evaporated ethanol and water and, (iii) heat for the ethanol azeotropic distillation from both: the fermentation broth outflow and the stream recovered from the offgas (data taken from [91]). The power requirements for cooling of bioreactor contents and compressed gases is not accounted for since the utility that would be used is cooling water at ambient temperature and thus its energetic burden is negligible.

22 $$U_{S} = \frac{{y_{D,i} \cdot F_{G,i} - y_{D,o} \cdot F_{G,o} }}{{y_{D,i} \cdot F_{G,i} }} \cdot 100\%$$

The MatLab codes developed within this study for simulating the CO and the H_2_/CO_2_ fermentation processes are available as Additional files 2 and 3, respectively.

#### Sensitivity analysis

Process performance is evaluated at different values of the constants governing the electron donor uptake kinetics (Eqs. 14 and 15), i.e, half-saturation constants ($K_{D}$) and the maximum specific uptake rates ($q_{D}^{ \text{max} }$). This analysis is made due to (i) uncertainty generated by the scarce information available in literature about these parameters, (ii) uncertainties associated with the methodologies used to fix these parameters (see section “Model validation”) and (iii) their paramount importance for the model reliability in quantitative terms.

Additionally, bioreactor performance is also assessed using different values of the mass transfer coefficients, the concentration of ethanol, the height of the liquid column and the gas hold-up. Specifically, 100% higher and 50% lower values of the mass transfer coefficients for CO, H_2_ and CO_2_ (as predicted by Eq. 16) are used. The range of possible $k_{L} a$ values was selected considering that: (i) the possible presence of surfactants that may hamper mass transfer by 70% at concentrations as low as 10 ppm [92]; (ii) the uncertain effect of ethanol concentration on mass transfer since it has been reported that ethanol at a wide range of concentrations may rise the gas hold-up by four times [86] while at 50 g L^−1^, the mass transfer coefficient would increase by 50% [93] and (iii) the proven ability of *C. ljungdahlii* and *C. carboxydivorans* [69, 74, 94, 95] to form biofilms which, if shaped into granules may enhance mass transfer by 30% due to intraparticle liquid circulation forced by pressure gradients caused by the circulation of bacteria inside the bioreactor [96].

Similarly, the maintenance of ethanol concentrations at 80 g L^−1^ are assessed considering that long-term adaptation experiments of *C. thermocellum* led to a 100% increase in its tolerance to both, ethanol and *n*-butanol [97], promoted in part by a change in the structural composition of its membrane.

Finally, disregarding possible conflicts with legislation and safety measures, the effect of an increased aspect ratio of the bubble column, form 3 to 10, is also assessed. Moreover, considering that 15 and 30% are regraded as standard values for large bubble column bioreactors to maintain high productivites [83], the effects of using a gas hold-up value of 30% on the process and the bioreactor performance are also assessed in the sensitivity analysis.

## Supplementary information

**Additional file 1.** Supplementary information about the equations used for the construction of the model and supporting information about examples given within the main document.

**Additional file 2.** MatLab codes for the simulation of CO fermentation.

**Additional file 3.** MatLab codes for the simulation of H_2_/CO_2_ fermentation.

## Data Availability

The MatLab codes created and used for the current study have been provided as additional files and are also available in the Zenodo repository.

## Latin letters

ATP: Adenosine triphosphate
$C$: Concentration in fermentation broth, mol m^−3^
${\text{CS}}$: Carbon source, i.e., CO or CO_2_
$D$: Electron donor of catabolism, i.e, CO, H_2_
${\mathcal{D}}$: Diffusivity in pure water, m^2^ s^−1^
*F*: Molar flow rate, mol s^−1^
*g*: Gravity’s acceleration, 9.8 m s^−1^
$H_{\text{mix}}$: Height of gas–liquid column the bioreactor, m
$k_{L} a$: Gas–liquid mass transfer coefficient, s^−1^
$K$: Half-saturation constant, mol m^−3^
$K_{I}$: CO inhibition constant, mol m^−3^
$$N_{\text{mix}}$$: Mixing number, 16 [91]
$n_{c}$: Number of mixing cells in the bioreactor
$$p$$: Pressure, Pa
$q$: Biomass specific production/consumption rate, mol Cmol_*x*_^−1^ h^−1^
$$R$$: Volumetric productivity, g_ethanol_ L^−1^ h^−1^
${\mathcal{R}}$: Ideal gas constant, 8.134 m^3^ Pa mol^−1^ K^−1^
$$T$$: Process temperature, K
$$t_{m}$$: Mixing time, s
$$U_{S}$$: Gas utilization, %
$v_{sG}^{c}$: Pressure-corrected superficial gas velocity, m s^−1^
$\dot{V}$: Volumetric flow rate, m^3^ s^−1^
WLP: Wood–Ljungdahl pathway
$y$: Molar fraction in gas phase
$Y_{x/CS}$: Biomass yield per mole of carbon source, Cmol_*x*_ mol_CS_^−1^

## Greek letters

$\gamma$: Degree of reduction, mol_*e*−_ mol^−1^
$\Delta G^{0}$: Standard Gibbs free energy change, kJ mol^−1^
$\Delta G_{\text{dis}}$: Gibbs free energy dissipation in anabolism, kJ mol^−1^
$\Delta G^{0\prime }$: Gibbs free energy change at physiological conditions, kJ mol^−1^
$\Delta H^{0}$: Standard enthalpy change, kJ mol^−1^
$$\varepsilon$$: Hold-up
$\mu$: Biomass growth rate, h^−1^
$\tau$: Diameter of bioreactor vessel, m
$\nu$: Stoichiometric coefficient; positive for products, negative for reactants

## Subscripts and superscripts

$${\text{an}}$$: Anabolism
$b$: Calculated at bottom of fermentor
$${\text{cat}}$$: Catabolism
$${\text{cons}}$$: Theoretically consumed
$${\text{et}}$$: Ethanol
$$e^{ - }$$: Electron
$$G$$: Gas
$i$: At gas inlet
$L$: Liquid fermentation broth
$${ \text{max} }$$: Maximum
$${\text{met}}$$: Metabolism
$o$: At bioreactor outlet
$$r$$: Reaction
$S$: Gas components, i.e, CO, H_2_, CO_2_
$t$: Calculated at top of fermentor
$$w$$: Water
$x$: Dry microbial biomass
$0$: Calculated at standard conditions, 101 kPa and 0 °C
