# Supplementary material for: Modeling ethanol production through gas fermentation: a biothermodynamics and mass transfer-based hybrid model for microbial growth in a large-scale bubble column bioreactor
Source: Biotechnol Biofuels. 2020 Mar 27;13:59. doi: 10.1186/s13068-020-01695-y (PMC7102449; doi:10.1186/s13068-020-01695-y)
Supplement: Supplementary file 1 — Additional file 1. Supplementary information about the equations used for the construction of the model and supporting information about examples given within the main document. [file 13068_2020_1695_MOESM1_ESM.docx]

**Additional file 1**

**Title:** Modeling ethanol production through gas fermentation: A biothermodynamics and mass transfer-based hybrid model for microbial growth in a large-scale bubble column bioreactor

**Authors:** Eduardo Almeida-Benalcázar^a,b,^*; Henk Noorman^b,c^; Rubens Maciel Filho^a^; John Posada-Duque^a^

^a^ Department of Product and Process Development, Faculty of Chemical Engineering, State University of Campinas, Av. Albert Einstein 500 - Cidade Universitária, 13083-852, Campinas – SP, Brazil.

^b^ Department of Biotechnology, Faculty of Applied Sciences, Delft University of Technology, Van der Maasweg 9, 2629 HZ, Delft, The Netherlands.

^c^ DSM Biotechnology Center, A. Fleminglaan 1, 2613 AX, Delft, The Netherlands.

**Corresponding author information:**

Department of Product and Process Development, Faculty of Chemical Engineering, State University of Campinas, Av. Albert Einstein 500 - Cidade Universitária, 13083-852, Campinas – SP, Brazil. Phone: +55(0)19996003377. Email: E.F.AlmeidaBenalcazar@tudelft.nl / ealmeidabenalc@feq.unicamp.br

1. **Data used for validation of threshold CO, H_2_ and CO_2_ concentrations**


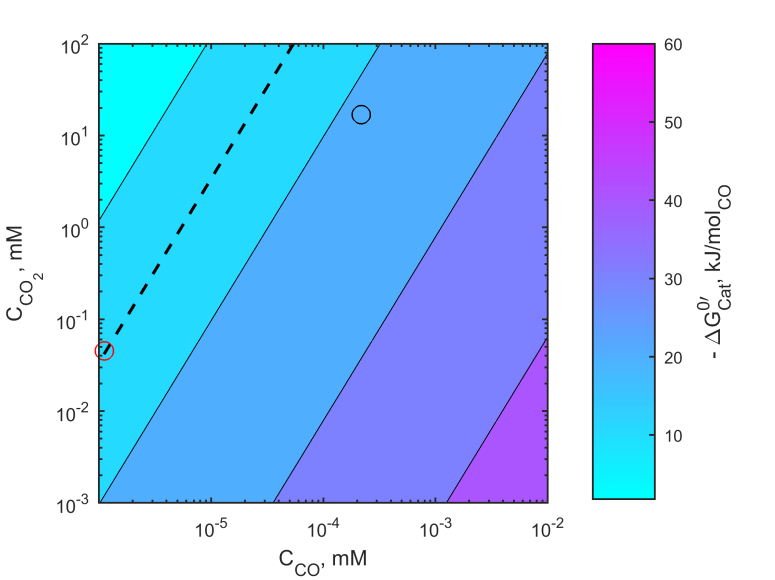


**Figure S1.** Gibbs free energy generation through independent CO catabolism for acetate production at 65 °C. The dashed lines indicate where ${\Delta G}_{cat}^{0'}$ = –15 kJ/mol_CS_. The black and red circles show the experimental data reported by [1] which were used for model validation

**
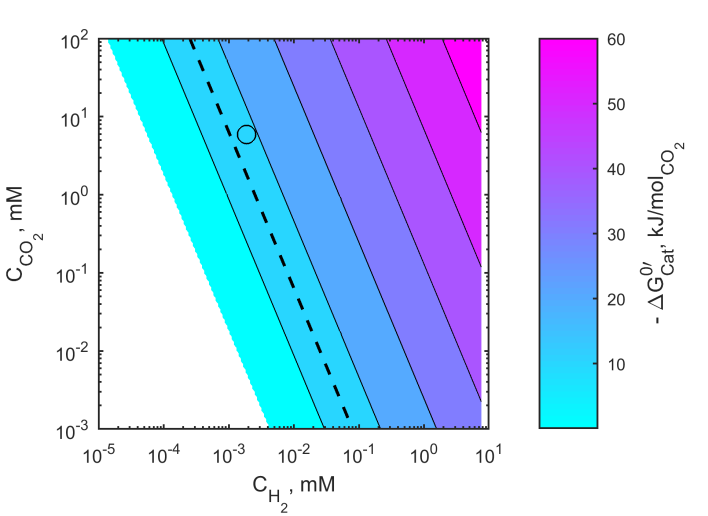
**

**Figure S2.** Gibbs free energy generation through independent H_2_/CO_2_ catabolism for acetate production at 30 °C. The dashed lines indicate where ${\Delta G}_{cat}^{0'}$ = –15 kJ/mol_CS_. The black circle shows the experimental data reported by [2] which was used for model validation

1. **Predicted biomass yields and data used for their validation**

**
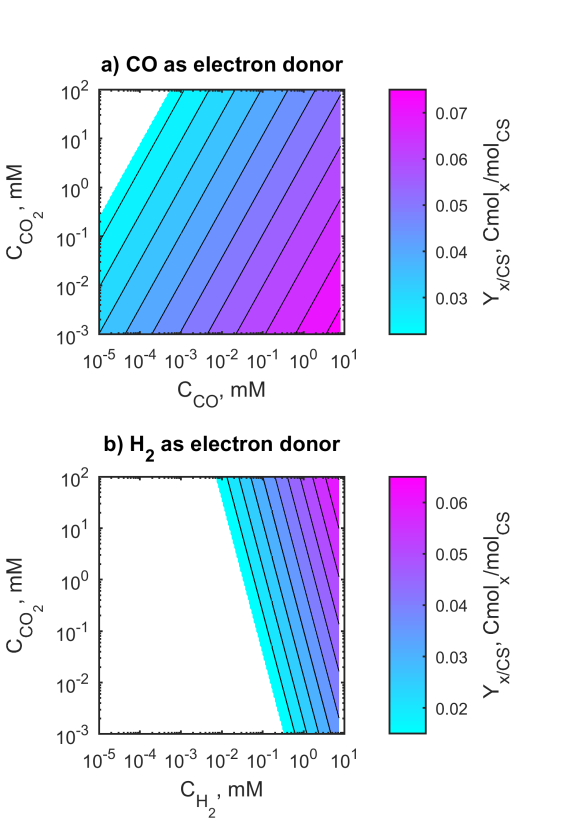
**

**Figure S3.** Biomass yields when acetogens produce ethanol for the fermentation of a) CO and b) H_2_/CO_2_

**Table SI1** Reported experimental data used for the validation of biomass yields

| **Experiment nr.** | **Reported q rates, mol/Cmol_x_/h** | | | | | | **Estimated parameters** | |
| --- | --- | --- | --- | --- | --- | --- | --- | --- |
|  | $\boldsymbol{\mu}$ | $\boldsymbol{q}_{\boldsymbol{CO}}$ | $\boldsymbol{q}_{\boldsymbol{H}_{\boldsymbol{2}}}$ | $\boldsymbol{q}_{\boldsymbol{CO}_{\boldsymbol{2}}}$ | $\boldsymbol{q}_{\boldsymbol{et}}$ | $\boldsymbol{q}_{\boldsymbol{HAc}}$ | $\frac{\boldsymbol{q}_{\boldsymbol{et}}}{\boldsymbol{q}_{\boldsymbol{et}}\boldsymbol{+}\boldsymbol{q}_{\boldsymbol{HAc}}}$ | $\boldsymbol{Y}_{\boldsymbol{x}/\boldsymbol{CS}}$ |
| *Data from Valgepea et al., 2017* [3] | | | | | | | | |
| 1 | 0.042 | 0.49 | 0.33 | 0.14 | 0.03 | 0.17 | 0.15 | 0.086 |
| 2 | 0.042 | 0.46 | 0.31 | 0.14 | 0.03 | 0.15 | 0.16 | 0.090 |
| 3 | 0.042 | 0.62 | 0.32 | 0.22 | 0.06 | 0.13 | 0.32 | 0.067 |
| 4 | 0.042 | 0.80 | 0.31 | 0.33 | 0.09 | 0.11 | 0.47 | 0.052 |
| 5 | 0.042 | 0.75 | 0.29 | 0.31 | 0.10 | 0.10 | 0.49 | 0.056 |
| *Data from Valgepea et al., 2018* [4] | | | | | | | | |
| 1 | 0.042 | 0.78 | 0.00 | 0.53 | 0.10 | 0.16 | 0.38 | 0.053 |
| 2 | 0.042 | 0.52 | 0.76 | 0.11 | 0.29 | 0.10 | 0.75 | 0.080 |
| *Data from Richter et al., 2013* [5] | | | | | | | | |
| 1 | 0.039 | 0.88 | 0.55 | 0.11 | 0.022 | 0.299 | 0.07 | 0.044 |

1. **Growth rate dependency on electron donor concentration**

**
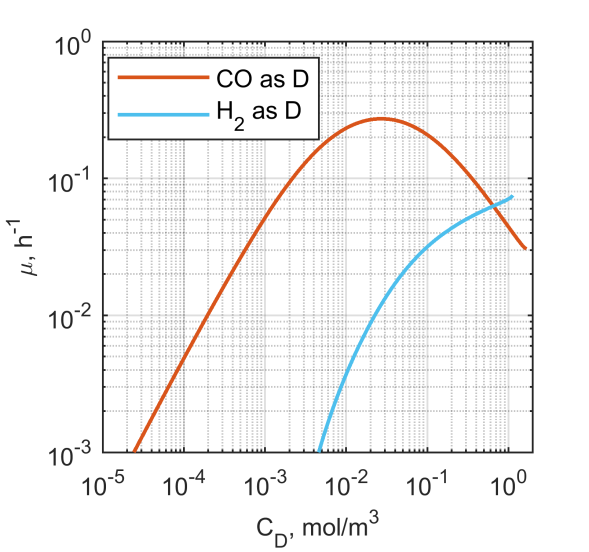
**

**Figure S4.** Growth rate dependency on the concentration of the electron donor

1. **Mass Balances and constraints used to guide convergence of the optimization algorithm**

**Table S2** List of mass balance equations and constraints used for convergence of the optimization algorithm

| *Equalities (the optimization algorithm ensures these constraints are equal to zero)* | | |
| --- | --- | --- |
| SI1 | Electron donor balance in the liquid phase  $0=k_{L}a_{D}\cdot\left( C_{D}^{*}-C_{D} \right)\cdot3600-q_{D}\cdot C_{x}-\dot{V}_{L,o}\cdot C_{D}$ | |
| SI2 | Electron donor balance in the gas phase  $0=F_{G,i}\cdot y_{D,i}-F_{G,o}\cdot y_{D,o}-k_{L}a_{D}\cdot\left( C_{D}^{*}-C_{D} \right)\cdot V_{L}\cdot3600$ | |
| SI3 | Overall CO_2_ balance (used in CO fermentation)  ${0=q}_{CO_{2}}\cdot C_{x}\cdot V_{L}-F_{G,o}\cdot y_{CO_{2},o}-\dot{V}_{L,o}\cdot C_{CO_{2},ml}$ | |
| SI4 | CO_2_ balance in the liquid phase (used in H_2_ fermentation)  $0=k_{L}a_{CO_{2}}\cdot\left( C_{CO_{2}}^{*}-C_{CO_{2}} \right)\cdot V_{L}\cdot3600-q_{CO_{2}}\cdot C_{x}\cdot V_{L}-\dot{V}_{L,o}\cdot C_{CO_{2}}$ | |
| SI5 | CO_2_ balance in the gas phase (used in H_2_ fermentation)  ${0=F}_{G,i}\cdot y_{CO_{2},i}-F_{G,o}\cdot y_{CO_{2},o}-k_{L}a_{CO_{2}}\cdot\left( C_{CO_{2}}^{*}-C_{CO_{2}} \right)\cdot V_{L}\cdot3600$ | |
| SI6 | Overall ethanol balance  $0=q_{et}\cdot C_{x}\cdot V_{L}\cdot3600-F_{G,o}\cdot y_{et,o}-\dot{V}_{L,o}\cdot C_{et}$ | |
| SI7 | Molar fraction summation in offgas  $0=1-y_{D,o}-y_{CO_{2},o}-y_{et,o}-y_{w,o}$ | |
| SI8 | Definition of the mean log molar gas flow rate | |
|  | $0=F_{G,ml}-\frac{\left\vert F_{G,o}-\frac{F_{G,i}}{1-\frac{p_{mix}}{p_{b}}} \right\vert}{\left\vert\ln\left( \frac{F_{G,o}}{\frac{F_{G,i}}{1-\frac{p_{mix}}{p_{b}}}} \right) \right\vert}$ | The correction $\frac{F_{G,i}}{1-\frac{P_{mix}}{P_{b}}}$ was applied to the gas flow rate at the fermentor inlet to account for the amount of evaporated water/ethanol mixture at the bottom of the fermentor |
| *Inequalities (the optimization algorithm ensures these constraints are always negative)* | | |
| SI9 | Thermodynamic feasibility constraint  $0>{\Delta G}_{cat}^{0'}$ | |
| SI10 | Positive liquid outflow rate constraint  ${0>-\dot{V}}_{L,o}$ | |
| SI11 | Overall water mass balance constraint (used in H_2_ fermentation)  ${0>q}_{w}\cdot C_{x}\cdot V_{L}\cdot3600-F_{G,o}\cdot y_{w,o}-\frac{V_{L,o}\cdot\left( 1-C_{et}\cdot MW_{et} \right)}{{MW}_{w}}$ | |

1. **Gas utilization inside the large syngas fermentor**

Figure SI5 represents the influence of the non-dimensional CO and H_2_ uptake rates on gas utilization inside the large-scale syngas fermentor.


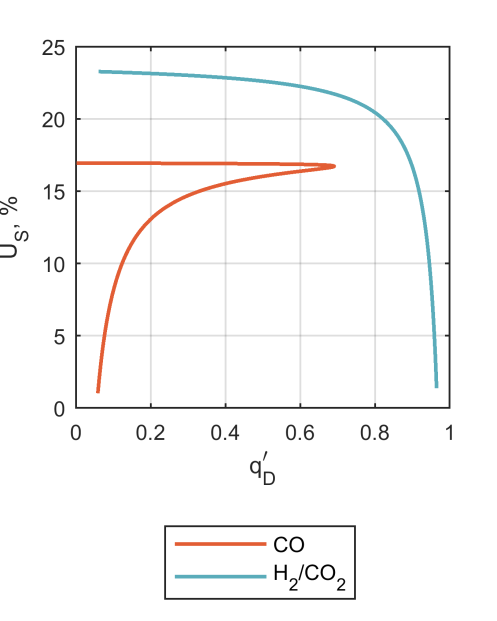


**Figure S5.** Relation between gas utilization inside the large-scale syngas fermentor and the non-dimensional uptake rate of the electron donor CO and H_2_

1. **List of equations used to estimate liquid-vapor equilibrium data for the ethanol-water mixture using the non-random two liquid model.**

The activity coefficients (eq SI12 – SI3) for ethanol (1) and water (2) are calculated from the composition (molar fractions) of the liquid phase. The composition of the vapor phase is finally estimated using Antoine’s equation (see eq SI18) and a modified Raoult’s law (see eq SI20). Figure SI6 shows vapor liquid equilibrium data generated by the non-random two liquid (NRTL) model.

**Table S3** List of equations used to estimate liquid-vapor equilibrium data for the ethanol-water mixture by the NRTL model

| **Equation** | | **Additional information** |
| --- | --- | --- |
| SI12 | $\ln\gamma_{1}^{a}={x_{2}}^{2}\cdot\left[ \tau_{21}\cdot\left( \frac{G_{21}}{x_{1}+x_{2}\cdot G_{21}} \right)^{2}+\frac{\tau_{12}\cdot G_{12}}{\left( x_{2}+x_{1}\cdot G_{12} \right)^{2}} \right]$ | Substance 1 is ethanol, substance 2 is water.  $\gamma_{1}^{a}$ is the activity coefficient of substance 1. |
| SI13 | $\ln\gamma_{2}^{a}={x_{1}}^{2}\cdot\left[ \tau_{12}\cdot\left( \frac{G_{12}}{x_{2}+x_{1}\cdot G_{12}} \right)^{2}+\frac{\tau_{21}\cdot G_{21}}{\left( x_{1}+x_{2}\cdot G_{21} \right)^{2}} \right]$ |  |
| SI14 | $\ln G_{12}=-\alpha_{12}\cdot\tau_{12}$ | $\alpha_{21}=\alpha_{21}=0.3$ |
| SI15 | $\ln G_{21}=-\alpha_{21}\cdot\tau_{21}$ |  |
| SI16 | $\tau_{12}=\frac{u_{12}-u_{22}}{R\cdot T}$ | $u_{12}-u_{22}=368.2$ J mol-1 [6] |
| SI17 | $\tau_{21}=\frac{u_{21}-u_{11}}{R\cdot T}$ | $u_{21}-u_{11}=4083.6$ J mol-1 [6] |
| SI18 | $\log\left( p_{1} \right)=A_{1}-\frac{B_{1}}{C_{1}+T}$ | \| **Component** \| **A** \| **B** \| **C** \| \| --- \| --- \| --- \| --- \| \| *1 (ethanol)* \| 7.24215 \| 1596.044 \| -46.655 \| \| *2 (water)* \| 7.11572 \| 1684.123 \| -43.568 \|   * Pressure is obtained in kPa, T is in K. data gathered from [7] |
| SI19 | $p_{mix}=p_{1}+p_{2}$ |  |
| SI20 | $y_{1}=p_{mix}x_{1}\gamma_{1}^{a}$ |  |

**
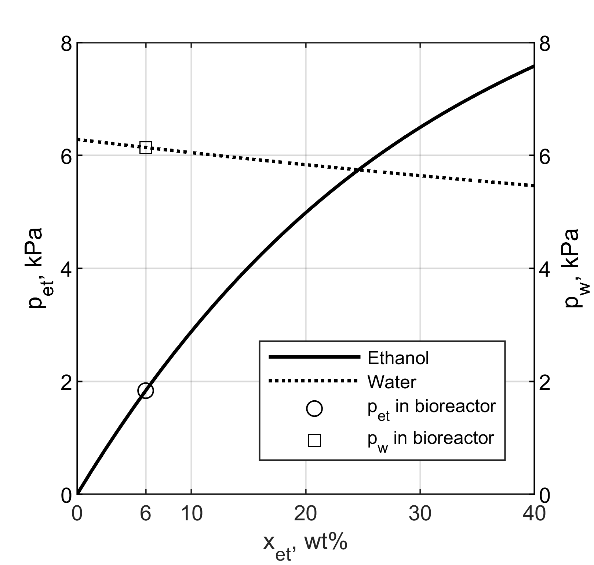
**

**Figure S6.** Equilibrium partial pressures for ethanol and water as functions of ethanol concentration in water. Ethanol partial pressures are shown at the left y-axis while water is shown at the right y-axis. The plot was generated using the NRTL model at 37 °C.

1. **List of equations used to calculate the gas flow rate flowing across the bioreactor from the pressure-corrected superficial gas velocity**

**Table S3** List of equations used to calculate the gas flow rate flowing across the bioreactor from the pressure-corrected superficial gas velocity

| **Equation** | | **Additional information** |
| --- | --- | --- |
| SI21 | $v_{sG}^{C}=v_{sG}^{0}\cdot p^{0}\cdot\left[ \frac{\log\left( \frac{p_{b}}{p_{t}} \right)}{p_{b}-p_{t}} \right]$ | superficial gas velocity is determined at normal conditions of temperature and pressure |
| SI22 | $v_{sG}^{0} =\frac{\dot{V}_{ml}^{0}}{A_{T}}$ | $A_{T}$ is the cross-sectional area of the bioreactor vessel. |
| SI23 | $\dot{V}_{ml}^{0}=\frac{\dot{V}_{ml}\cdot p_{ml}\cdot273.15}{101325\cdot T}$ | $p_{ml}$ is the log mean pressure inside the bioreactor. It is also assumed that the gas will behave as an ideal gas |
| SI24 | $\dot{V}_{ml}=\frac{F_{ml}\cdot R\cdot1000\cdot T}{p_{ml}}$ |  |

1. **Equations used for calculation of energy requirements**

**Table S4** List of equations used for calculation of energy requirements

| *Power for gas compression* | |
| --- | --- |
| SI25 | $P_{C,i}=\left( \frac{\Upsilon_{i}}{\Upsilon_{i}-1} \right) \cdot p_{atm}\cdot\dot{V}_{G,stoich}\cdot\left[ \left( \frac{p_{b}}{p_{atm}} \right)^{\left( \frac{\Upsilon_{i}-1}{\Upsilon_{i}} \right)}-1 \right]\cdot\frac{1}{0.7}$ |
| SI26 | $\Upsilon_{i}=\frac{{c_{P}}_{G,i}}{{c_{V}}_{G,i}}$ |
| SI27 | $\dot{V}_{G,stoich}=\frac{\left( \frac{q_{D}\cdot C_{x}\cdot V_{L}}{y_{D,i}\cdot3600} \right)\cdot R\cdot T}{p_{atm}}$ |
| SI28 | $P_{C,o}=\left( \frac{\Upsilon_{o}}{\Upsilon_{o}-1} \right) \cdot p_{t}\cdot\dot{V}_{G,o}\cdot\left[ \left( \frac{p_{b}}{p_{t}} \right)^{\left( \frac{\Upsilon_{o}-1}{\Upsilon_{o}} \right)}-1 \right]\cdot\frac{1}{0.7}$ |
| SI29 | $\Upsilon_{o}=\frac{{c_{P}}_{G,o}}{{c_{V}}_{G,o}}$ |
| *Power for reactor cooling* | |
| SI30 | $P_{cool}={\Delta H}_{met}^{T}\cdot\left[ k_{L}a_{D}\cdot\left( C_{D}^{*}-C_{D} \right){\cdot V}_{L} \right]+\left( \frac{p_{mix}\cdot F_{G,o}}{p_{t}} \right)\left[ \lambda_{w}^{T}\cdot\left( \frac{y_{w,o}}{y_{w,o}+y_{et,o}} \right)+\lambda_{et}^{T}\cdot\left( \frac{y_{et,o}}{y_{w,o}+y_{et,o}} \right) \right]$ |
| SI31 | ${\Delta H}_{met}^{T}={\Delta H}_{met}^{0}+\int_{298.15}^{T} {c_{P}}_{m}dT$ |
| SI32 | ${c_{P}}_{m}=\sum_{j=1}^{n} \nu_{j}^{met}\cdot{c_{P}}_{j}$ |
| *Power for condensation of evaporated water/ethanol mixture* | |
| SI33 | $P_{cond}=\left[ \lambda_{w}^{-6℃}\cdot\left( \frac{y_{w,o}}{y_{w,o}+y_{et,o}} \right)+\lambda_{et}^{-6℃}\cdot\left( \frac{y_{et,o}}{y_{w,o}+y_{et,o}} \right) \right]\cdot F_{G,o}\cdot\left( \frac{p_{mix}}{p_{t}} \right)\cdot\left( \frac{1}{3.7} \right)$ |
| *Power for distillation of ethanol in offgas condensate* | |
| SI34 | $P_{dis,G}=22.194\cdot\left( 100\cdot x_{et}^{G} \right)^{-0.794}\cdot f_{et}^{G}$ |
| SI35 | $f_{et}^{G}=\frac{y_{et,o}\cdot F_{G,o}\cdot3600}{q_{et}\cdot C_{x}\cdot V_{L}}$ |
| SI36 | $x_{et}^{G}=\frac{\left( \frac{y_{et,o}}{y_{w,o}+y_{et,o}} \right)\cdot{MW}_{et}}{\left[ \left( \frac{y_{et,o}}{y_{w,o}+y_{et,o}} \right)\cdot{MW}_{et}+\left( \frac{y_{w,o}}{y_{w,o}+y_{et,o}} \right)\cdot{MW}_{w} \right]}$ |
| *Power for distillation of ethanol in fermentation broth* | |
| SI37 | $P_{dis,L}=22.194\cdot\left( 100\cdot x_{et}^{L} \right)^{-0.794}\cdot f_{et}^{L}$ |
| SI38 | $f_{et}^{L}=\frac{C_{et}\cdot F_{L,o}\cdot3600}{q_{et}\cdot C_{x}\cdot V_{L}}$ |
| SI39 | $x_{et}^{L}=\frac{C_{et}\cdot MW_{et}}{1000000}$ |

**Table S5** Nomenclature table

| **Abbreviations** | | **Greek** | |
| --- | --- | --- | --- |
| $C$ | Concentration in fermentation broth, mol m^-3^ | $\Upsilon$ | Heat capacity ratio |
| $C^{*}$ | Saturation concentration, mol m^-3^ | $\lambda$ | Latent heat, kJ mol^-1^ |
| $c_{P}$ | Heat capacity at constant pressure, kJ mol^-1^ K^-1^ | **Subscripts** | |
| $c_{V}$ | Heat capacity at constant volume, kJ mol^-1^ K^-1^ | $atm$ | Atmospheric |
| $f$ | Fraction of overall production, mol (mol_et_ + mol_HAc_)^-1^ | $b$ | Fermentor bottom |
| $F$ | Molar flow rate, mol s^-1^ | $cat$ | Catabolism |
| ${\Delta G}^{0'}$ | Gibbs free energy change at physiological conditions, kJ mol^-1^ | $cond$ | Condensation |
|  |  | $cool$ | Cooling of fermentor contents |
| ${\Delta H}^{0}$ | Standard enthalpy change, kJ mol^-1^ | $C$ | Compression |
| ${\Delta H}^{T}$ | Enthalpy change at process temperature, kJ mol^-1^ | $dis$ | Distillation |
|  |  | $D$ | Electron donor of catabolism *i.e.* CO, H_2_ |
| $k_{L}a$ | Gas-liquid mass transfer coefficient, s^-1^ | $et$ | Ethanol |
| $MW$ | Molar mass, g mol^-1^ | $G$ | Gas |
| $P$ | Specific power consumption, kJ kg_ethanol_^-1^ | $i$ | At syngas inlet |
| $p$ | Absolute pressure, Pa | $L$ | Fermentation broth |
| $p_{mix}$ | Vapor pressure of water-ethanol mixture, Pa | $m$ | Temperature averaged |
| $q$ | Biomass specific production/consumption rate, mol Cmol_x_^-1^ h^-1^ | $met$ | Metabolism |
|  |  | $mix$ | Water-ethanol mixture at equilibrium with fermentation broth |
| $R$ | Ideal gas constant, 8.134 m^3^ Pa mol^-1^ K^-1^ |  |  |
| $T$ | Fermentation temperature, K | $ml$ | Logarithmic mean |
| $V$ | Volume, m^3^ | $o$ | At syngas outlet |
| $\dot{V}$ | Volumetric flow rate, m^3^ s^-1^ | $stoich$ | Stoichiometrically needed |
| $x$ | Mass fraction in ethanol-water mixture | $t$ | Fermentor top |
| $y$ | Molar fraction in gas phase | $w$ | water |
|  |  | $x$ | Dry microbial biomass |
|  |  | **Superscripts** | |
|  |  | $L$ | At the liquid phase |
|  |  | $G$ | At the gas phase |

1. **References**

[1] Henstra AM. CO metabolism of *Carboxydothermus hydrogenformans* and *Archaeoglobus fulgidus*. PhD Thesis. Wageningen University, 2006.

[2] Poehlein A, Schmidt S, Kaster A-K, Goenrich M, Vollmers J, Thürmer A, et al. An ancient pathway combining carbon dioxide fixation with the generation and utilization of a sodium ion gradient for ATP synthesis. PLoS ONE 2012;7:e33439. https://doi.org/10.1371/journal.pone.0033439.

[3] Valgepea K, de Souza Pinto Lemgruber R, Meaghan K, Palfreyman RW, Abdalla T, Heijstra BD, et al. Maintenance of ATP homeostasis triggers metabolic shifts in gas-fermenting acetogens. Cell Syst 2017;4:505-515.e5. https://doi.org/10.1016/j.cels.2017.04.008.

[4] Valgepea K, de Souza Pinto Lemgruber R, Abdalla T, Binos S, Takemori N, Takemori A, et al. H_2_ drives metabolic rearrangements in gas-fermenting *Clostridium autoethanogenum*. Biotechnol Biofuels 2018;11:55. https://doi.org/10.1186/s13068-018-1052-9.

[5] Richter H, Martin M, Angenent L. A Two-Stage Continuous Fermentation System for Conversion of Syngas into Ethanol. Energies 2013;6:3987–4000. https://doi.org/10.3390/en6083987.

[6] Gmehling J, Onken U, Arlt W, editors. Vapor-liquid equilibrium data collection: tables and diagrams of data for binary and multicomponent mixtures up to moderate pressures; constants of correlation equations for computer use. Pt. 1: Aqueous-organic systems. 2. ed., 3. printing. Frankfurt Am Main: Dechema; 1991.

[7] Kurihara K, Minoura T, Takeda K, Kojima K. Isothermal Vapor-Liquid Equilibria for Methanol + Ethanol + Water, Methanol + Water, and Ethanol + Water. J Chem Eng Data 1995;40:679–84. https://doi.org/10.1021/je00019a033.
